# *Solanum pimpinellifolium* exhibits complex genetic resistance to *Pseudomonas syringae* pv. *tomato*

**DOI:** 10.3389/fpls.2024.1416078

**Published:** 2024-10-23

**Authors:** Jana A. Hassan, Nathan Diplock, Ilea J. Chau-Ly, Jamie Calma, Elizabeth Boville, Steven Yee, Taylor M. Harris, Jennifer D. Lewis

**Affiliations:** ^1^ Department of Plant and Microbial Biology, University of California, Berkeley, Albany, CA, United States; ^2^ Plant Gene Expression Center, United States Department of Agriculture, Albany, CA, United States

**Keywords:** bacterial speck, genetic diversity, quantitative trait loci (QTL), plant breeding, Pseudomonas syringae, Solanum pimpinellifolium

## Abstract

*Pseudomonas syringae pv. tomato* (*Pst*) is the causal agent of bacterial speck disease in tomatoes. The *Pto/Prf* gene cluster from *Solanum pimpinellifolium* was introgressed into several modern tomato cultivars and provided protection against *Pst* race 0 strains for many decades. However, virulent *Pst* race 1 strains that evade *Pto*-mediated immunity now predominate in tomato-growing regions worldwide. Here we report the identification of resistance to a *Pst* race 1 strain (*Pst*19) in the wild tomato accession *S. pimpinellifolium* LA1589 (hereafter LA1589), using our rapid high-throughput seedling screen. LA1589 supports less bacterial growth than cultivars, and does not exhibit a hypersensitive response to *Pst*19. We tested an existing set of 87 Inbred Backcross Lines (IBLs) derived from a cross between susceptible *Solanum lycopersicum* E-6203 and *Solanum pimpinellifolium* LA1589 for resistance to *Pst*19. Using single-marker analysis, we identified three genomic regions associated with resistance. Bacterial growth assays on IBLs confirmed that these regions contribute to resistance *in planta*. We also mapped candidate genes associated with resistance in a cross between the *Solanum lycopersicum* var. *lycopersicum* cultivar Heinz BG-1706 and *S. pimpinellifolium* LA1589. By comparing candidates from the two mapping approaches, we were able to identify 3 QTL and 5 candidate genes in LA1589 for a role in resistance to *Pst*19. This work will assist in molecular marker-assisted breeding to protect tomato from bacterial speck disease.

## Introduction

Bacterial speck disease of tomato, caused by *Pseudomonas syringae pv. tomato* (*Pst*), is a persistent global problem, affecting both the marketability and yield of fresh-market and processing tomatoes (Yunis et al., 1980; Kunkeaw et al., 2010). *Pst* is primarily a foliar pathogen that grows as an epiphyte on the plant surface, and gains entry through the stomata to proliferate in the plant apoplast (Hirano and Upper, 2000; Xin et al., 2018). Plants defend themselves against pathogens through pattern-triggered immunity (PTI), where conserved microbial molecules are recognized, and effector-triggered immunity (ETI), where specific effectors are recognized. PTI occurs at the surface of the plant cell, where pattern recognition receptor proteins (PRRs) recognize highly conserved pathogen-associated molecular patterns (Couto and Zipfel, 2016). As a result of PTI, the host upregulates a variety of defense responses, including reinforcement of the cell wall, production of reactive oxygen species, and stomatal closure (Couto and Zipfel, 2016; DeFalco and Zipfel, 2021). To overcome PTI, bacteria utilize a type III secretion system to translocate effector proteins directly into the plant cell, where they target components of PTI and promote bacterial virulence (Lewis et al., 2009; Block and Alfano, 2011; Schreiber et al., 2021a). ETI occurs upon recognition of effectors by nucleotide-binding-site leucine-rich repeat receptor (NLRs, also called Resistance (R) proteins), which triggers a rapid and robust secondary immune response (DeYoung and Innes, 2006; Jones et al., 2016; Schreiber et al., 2016). This is often associated with localized cell death at the site of infection, termed the hypersensitive response (HR) (Heath, 2000). In addition to the gene-for-gene resistance often associated with ETI, plants can also exhibit quantitative disease resistance (QDR), in which multiple loci partially contribute to disease resistance (Poland et al., 2009; Boyd et al., 2013; French et al., 2016). These loci can include genes other than PRRs or NLRs. QDR typically results in less disease, rather than an absence of disease, and can be affected by environmental conditions (French et al., 2016). Since QDR is conferred by multiple genes, it is less likely to be overcome by pathogens, and is therefore very useful in breeding programs (Poland et al., 2009).

Historically, bacterial speck of tomato was primarily caused by *Pst* race 0 strains, which translocate a suite of type III secreted effector proteins (T3SEs) that includes AvrPto and AvrPtoB (Pedley and Martin, 2003). In the 1930s, the *Pto/Prf* gene cluster was introgressed from the wild tomato species *Solanum pimpinellifolium* into processing tomato cultivars to provide protection against bacterial speck disease caused by *Pst* race 0 strains (Pilowsky and Zutra, 1982; Kerr and Cook, 1983; Pitblado and MacNeill, 1983; Pedley and Martin, 2003). Prf is a NLR that interacts with Pto, an intracellular serine/threonine protein kinase. The Prf/Pto complex recognizes effectors AvrPto and AvrPtoB through direct interaction, and triggers ETI (Ronald et al., 1992; Martin et al., 1993; Salmeron et al., 1996; Tang et al., 1996). However, the *Pto/Prf* gene cluster does not recognize *Pst* race 1 strains, which have emerged as the prevalent strains throughout the world (Kunkeaw et al., 2010; Cai et al., 2011; Valenzuela et al., 2022). *Pst* race 1 strains are differentiated from *Pst* race 0 strains by the loss, mutation, or post-transcriptional down-regulation of AvrPto and AvrPtoB, which abrogates recognition by Pto/Prf (Lin et al., 2006; Almeida et al., 2009; Kunkeaw et al., 2010).

Wild relatives of crop plants are excellent sources of natural genetic diversity for traits of interest, including pathogen resistance (Zamir, 2001). Several studies in adult plants have identified resistance to *Pst* race 1 strains in wild relatives of tomato. The wild tomato accession *S. habrochaites* LA1777 demonstrates resistance to *Pst*A9, a *Pst* race 1 isolate found in California, and four quantitative trait loci (QTL) associated with resistance were identified using a population of introgression lines (Thapa et al., 2015). Another *S. habrochaites* accession, LA2109, demonstrates resistance to the *Pst* race 1 strain T1 (*Pst*T1) (Bao et al., 2015). Two QTL and a candidate gene for resistance were identified using a mapping-by-sequencing approach in LA2109. Recently, researchers identified the NLR Ptr1, which is able to detect the activity of the effector AvrRpt2 and trigger resistance to *Pst*T1 in *S. lycopersicoides* LA2951 (Mazo-Molina et al., 2019, Mazo-Molina et al., 2020).

To rapidly screen for resistance to *P. syringae* in wild tomato accessions, we developed a high-throughput seedling-based flood assay which faithfully recapitulates adult phenotypes (Hassan et al., 2017, Hassan et al., 2020). We demonstrated that seedlings of cultivars containing the *Pto/Prf* cluster recognize *Pst*DC3000, resulting in seedling survival, reduced bacterial growth, a hypersensitive response, and a rapid increase in ion leakage (Hassan et al., 2017). Seedlings of cultivars that lack the *Pto/Prf* cluster are susceptible to *Pst*DC3000, and support high levels of bacterial growth with eventual death of the seedlings (Hassan et al., 2017). Using this screen, we identified two additional wild tomato accessions which demonstrate strong resistance to *Pst*19, *S. neorickii* LA1329 and *S. habrochaites* LA1253 (Hassan et al., 2017). *Pst*19 is a hypervirulent strain of *P. syringae* closely related to *Pst*T1 (Kunkeaw et al., 2010). *S. neorickii* LA1329 displays genetically complex resistance to *Pst*19 in both seedlings and adult plants (Hassan et al., 2017).

In this study, we identified a wild tomato line, *S. pimpinellifolium* LA1589, which exhibits resistance to *Pst*19 in both seedlings and adults. Although many *S. pimpinellifolium* accessions possess the *Pto*/*Prf* gene cluster (Pitblado and MacNeill, 1983; Pedley and Martin, 2003), we found that resistance to *Pst*19 was not dependent on Pto/Prf. Additionally, we found that LA1589 does not exhibit an HR or levels of bacterial growth that are characteristic of ETI, suggesting it has QDR to *Pst*19. We used a pre-existing population of *S. pimpinellifolium* LA1589 recombinant inbred backcross lines (IBLs) (Doganlar et al., 2002), in parallel with an F_2_ mapping population derived from LA1589 and the susceptible cultivar Heinz BG-1706 to identify genomic regions and candidate genes associated with resistance to *Pst*19. The identification of these regions could facilitate the future breeding of tomato varieties resistant to bacterial speck caused by *Pst* race 1 strains.

## Materials and methods

### Plant materials and growth conditions

Tomato seeds were sterilized in 50% bleach for 30 min. After sterilization, the seeds were rinsed five times with sterile nanopure H_2_O and then plated or sown out on soil. Seeds for plate experiments were germinated on 100 x 25 mm plates containing sterile 0.5 X Murashige and Skoog (MS) basal salts and 0.8% agar. Seeds used for soil experiments were planted in Sunshine Mix#1/LC1 (Sun Gro Horticulture Canada Ltd.) supplemented with 15:9:12 fertilizer. Seeds on plates or soil were stratified at 4°C for 3 days to synchronize germination. Plants were grown in a growth chamber under a constant temperature of 22°C and 16 h of light (200-220 µE m^-2^ s^-1^) and 8 h of darkness. The following tomato accessions were obtained from the Tomato Genetics Resource Center (tgrc.ucdavis.edu): *S. lycopersicum* MoneyMaker-*PtoS*, *S. lycopersicum* MoneyMaker-*PtoR*, *S. lycopersicum* LA3342 (RioGrande-*PtoR*), *S. lycopersicum* LA3343 (RioGrande-*PtoS*), *S. lycopersicum* LA4345 (Heinz BG-1706), *S. pimpinellifolium* LA1589 (also called PI407545), *S. lycopersicum* LA4024 (E-6203) and recombinant Inbred Backcrossed Lines (IBL) *S. pimpinellifolium* LA4139 – LA4229 (Doganlar et al., 2002). 100 IBLs were identified based on uniform genome coverage and map resolution (Doganlar et al., 2002), and 87 lines were available from the Tomato Genetics Resource Center.

### *P. syringae* strains, culture conditions and infection assays

*P. syringae* pv. *tomato* strains were grown in King’s broth (KB) media. Antibiotics were used at the following concentrations: 50 µg/mL rifampicin dissolved in dimethylformamide, 50 µg/mL cycloheximide dissolved in ethanol.

For seedling hypersensitive response (HR) assays, *Pst*19 was resuspended to an optical density at 600 nm (OD_600_) of 0.1 (approximately 5 x 10^7^ CFU/ml) and pressure infiltrated into both cotyledons. The HR was scored 16-20 h post-infiltration. For the seedling flood assay, *Pst*19 and *Pst*DC3000 were resuspended to an OD_600_ of 0.1 and then serially diluted in 10 mM MgCl_2_ to a final OD_600_ of 0.0075 and 0.005, respectively with 0.015% Silwet L-77 (Hassan et al., 2020). Ten-day-old seedlings were flooded for 3 min with 6 mL of inoculum or 10 mM MgCl_2_. Seedlings were phenotyped for disease or resistance 10-14 days after flooding. For bacterial growth assays in seedlings, seedlings were flooded as described above and four days later, one cotyledon was removed, surface sterilized in 70% ethanol for 10 s and rinsed in nanopure H_2_O for 10 s. Each cotyledon was blotted, individually weighed and homogenized in 10 mM MgCl_2_. Homogenized lysate was plated on KB with 50 µg/mL rifampicin and 50 µg/mL cycloheximide for colony counting. Cycloheximide prevents fungal contamination. Colony counts for seedlings were normalized to 0.1 g of tissue for cotyledons (Hassan et al., 2017).

For infection assays in adult plants, *P. syringae* was resuspended in 10 mM MgCl_2_ to an OD_600_ of 0.2 with 200 µL/L of Silwet L-77. Adult plants at the 4-6 leaf stage were inverted, then submerged and swirled in the inoculum for 30 s. Infected plants were incubated in a growth chamber under a humidity dome for 1-2 days at which time the dome was removed. Adult bacterial growth assays were performed 5 days after infection on abaxial leaflets of the 4^th^ leaf. Tissue was sterilized as described above for seedlings. A total of 1 cm^2^ tissue (four disks) was harvested from infected leaves, ground in 10 mM MgCl_2_, and plated as described for seedlings. Plants were re-incubated and phenotyped 5-7 days after infection.

### Screening and analysis of IBL population

IBLs were screened in the seedling flood assay as described above, and the number of surviving or deceased individuals was counted. Selected IBLs were tested for bacterial growth and symptoms in adult plants as described above.

Single-marker analysis was performed on the IBLs in the seedling flood assay using the Wilcoxon Mann-Whitney test, a nonparametric counterpart of the t-test because this test does not assume the probability distribution of the quantitative trait. For each marker, survival rates of seedlings homozygous for either parental allele were compared to determine significant differences. Marker loci were determined to be highly significantly associated with resistance at p<0.01, significantly associated with resistance at p<0.05 and suggestive of resistance at p<0.1. QTL mapping intervals were defined as regions including marker loci significantly associated with resistance, whose boundaries were defined at the first instance of an adjacent nonsignificant marker locus.

### Generation and screening the F2 Heinz-BG1706 x *S. pimpinellifolium* LA1589 mapping population

Heinz BG-1706 and a *Pst*19*-*resistant S*. pimpinellifolium* LA1589 individual were crossed. Seedlings from the F2 segregating population were grown on plates and flooded using the seedling flood assay protocol (Hassan et al., 2020). Each seedling was labeled with a unique number. Two days after flooding, the tip of one cotyledon from each seedling was snipped, frozen in liquid N_2_ and stored at -80°C. Plates were resealed and seedlings re-incubated in the growth chamber at 22°C. Highly susceptible seedlings were phenotyped 7-9 days after infection and strongly resistant seedlings were phenotyped 10 days after infection. Chi-squared goodness of fit test followed by Yates correction was performed to test the hypothesis of a Mendelian segregation ratio of 3 (susceptible): 1 (resistance) in the F2 population.

### Nucleic acid isolation, library construction, and sequencing

Total genomic DNA was extracted from pools of strongly resistant LA1589 or highly susceptible LA1589 F2 individuals, respectively using the Puregene Core Kit A (Qiagen Inc.). Libraries were constructed at the Functional Genomics Library (FGL), a QB3-Berkeley Core Research Facility at UC Berkeley. A S220 Focused-Ultrasonicator (Covaris) was used to fragment genomic DNA to 1-6000 bp and library preparation was performed using the KAPA Hyper Prep kit for DNA (KK8504). Truncated universal stub adaptors were used during PCR amplification to complete the adapters and to enrich the libraries for adapter-ligated fragments. Samples were checked for quality on an AATI Fragment Analyzer. Samples were then transferred to the Vincent J. Coates Genomics Sequencing Laboratory (GSL), another QB3-Berkeley Core Research Facility at UC Berkeley, where Illumina sequencing library molarity was measured with quantitative PCR with the Kapa Biosystems Illumina Quant qPCR Kits on a BioRad CFX Connect thermal cycler. Libraries were then pooled evenly by molarity and sequenced on an Illumina NovaSeq6000 150PE S4 flowcell, generating 10 Gb minimum of data per sample. Raw sequencing data was converted into fastq format, sample specific files using Illumina bcl2fastq2 software.

### Quality control, alignment, and variant calling

Paired-end Illumina read quality was checked with FastQC v0.11.7 (Andrews, 2010) and read fastq files were trimmed using Cutadapt v2.4 (Martin, 2011) to remove TruSeq adapter sequences 5’ACACTCTTTCCCTACACGACGCTCTTCCGATCT3’ and 5’GATCGGAAGAGCACACGTCT3’. BWA-MEM v0.7.17 (Li and Durbin, 2009; Li, 2013) was used to align paired-end reads from the pool of strongly resistant LA1589 individuals or the pool of highly susceptible LA1589 individuals to: 1) the SL4.0 Heinz BG-1706 reference build (Su et al., 2021), 2) the *Solanum pimpinellifolium* LA2093 genome (Wang et al., 2020) and, 3) the *Solanum pimpinellifolium* LA1589 PacBio genome (Alonge et al., 2020). Variants in candidate genes were identified by mapping to Heinz BG-1706, LA1589 or LA2093. Heinz BG-1706 variants were identified using Wgsim (Li, 2021) to simulate 10^8^ Illumina reads from the Heinz BG-1706 genome (SL4.0 build). Simulated reads from Heinz BG-1706 were aligned to the LA1589 Pacbio reference genome (Alonge et al., 2020) or the LA2093 reference genome (Wang et al., 2020) using BWA-MEM v0.7.17. BAM files from the alignments were sorted using SAMtools v1.8 (Li et al., 2009). BCFtools v.16 was used to perform variant calling. Low-quality variants with read depths of less than 10 were filtered out.

SnpSift was used to further filter candidate genes for homozygous variants (Cingolani et al., 2012a). Variants with a maximum fraction of reads supporting an indel of less than 0.1 were filtered out (http://www.htslib.org/doc/). SnpEff databases were built using SL4.1, LA2093 PacBio and LA1589 Pacbio annotations (Alonge et al., 2020; Wang et al., 2020; Su et al., 2021). SnpEff was used to predict the effects of variants on the translation of annotated genes (Cingolani et al., 2012b). Genes were considered candidates for resistance or susceptibility if a) they were within the mapping intervals from the IBL screen and b) had variants for which the SNPEff putative variant impact was classified as high or moderate impact. SNPEff predicts high impact variants to have a disruptive effect on the protein and moderate impact variants to have changes that are not disruptive but may alter the effectiveness of the protein.

LA1589 orthologs of Heinz BG-1706 or LA2093 candidates were identified using Reciprocal best Basic Local Alignment Search Tool (BLAST version 2.2.31+). Genome-to-genome protein sequence comparisons were made between the query genome (LA1589) and the database genome (Heinz BG-1706 SL4.1), as well as the reciprocal sequence comparisons. The LA1589 and LA2093 proteins were compared in the same manner. A maximum E-value threshold of 1x10^-6^ was used with Smith-Waterman alignment (Ward and Moreno-Hagelsieb, 2014). Top best hits for query proteins were sorted and compared to top best hits in the reciprocal direction. If no orthologs were identified, then closest homologs were identified using BLAST version 2.2.31+ for high bit-score and low e-values (Pearson, 2013).

## Results

### *S. pimpinellifolium* LA1589 exhibits resistance to *Pst*19 in both tomato seedlings and adult plants

**Figure 1:**
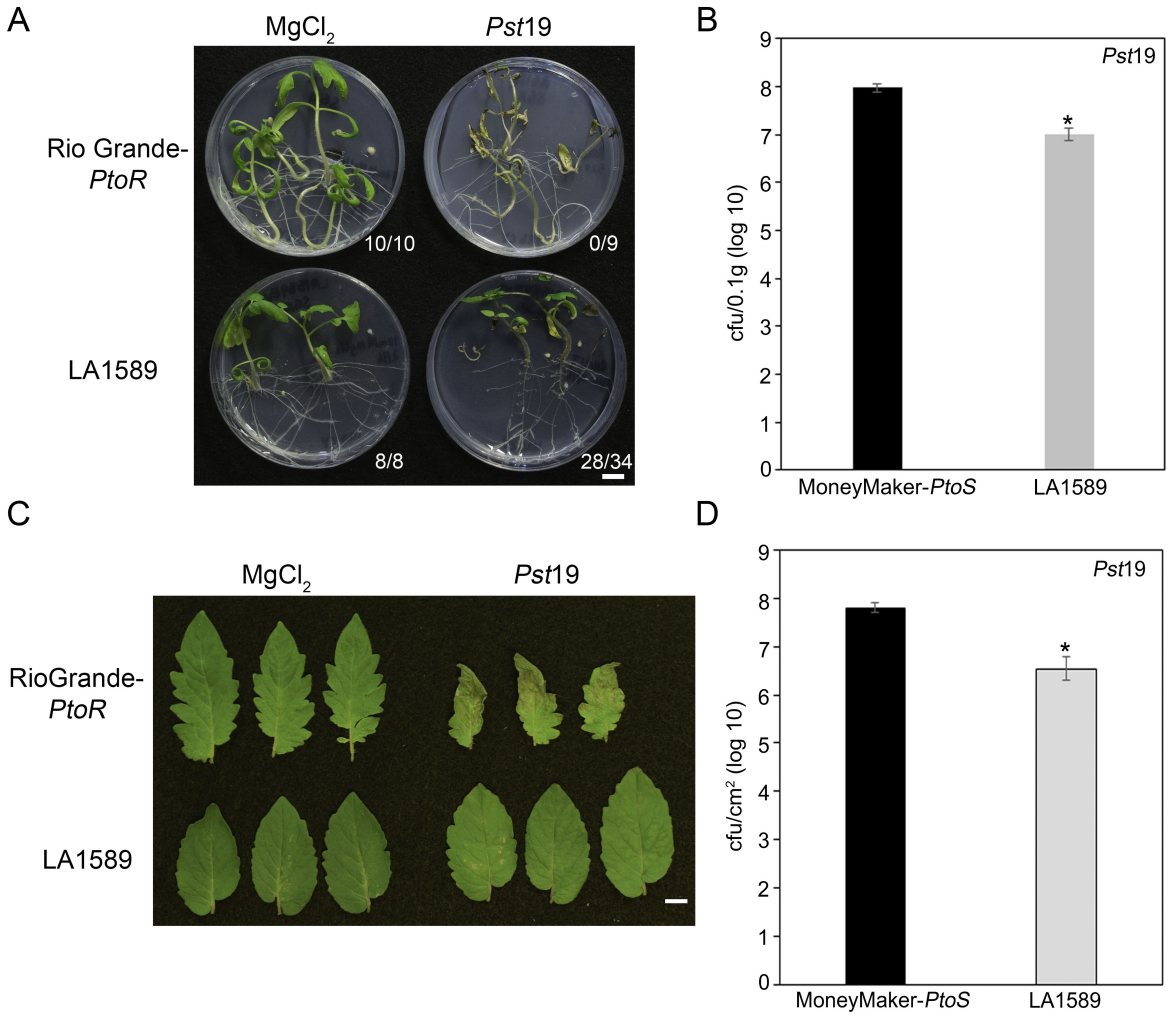
Both LA1589 seedlings and adults exhibit qualitative and quantitative resistance to *Pst*19. **(A)** Phenotypic resistance or disease symptoms in Rio Grande-*PtoR* or LA1589 tomato seedlings 10-14 days after being flooded with *Pst*19 at an OD_600_ of 0.0075. The number of surviving plants is indicated over the total number of tested plants. **(B)** Bacterial counts were determined 4 days post-infection (dpi) on MoneyMaker-*PtoS* (n=10) and LA1589 (n=23) seedlings and normalized to 0.1 g of tissue. The asterisk above the bar indicates statistical difference as determined by a one-factor ANOVA using a GLM procedure (p<0.05). The error bars indicate the standard error. The experiment was repeated 3 times with similar results. **(C)** Rio Grande-*PtoR* or LA1589 were grown to the 4-6 leaf stage, and dip-inoculated with a bacterial suspension at an OD_600_ of 0.2. Leaves were photographed 7 days after infection. The scale bar indicates 1 cm. **(D)** Bacterial counts were determined 5 days post infection on LA1589 (n=10) and MoneyMaker-*PtoS* (n=5) plants. The asterisk above the bar indicates statistical difference as determined by a one-factor ANOVA using a GLM procedure (p<0.05). The error bars indicate the standard error. The experiment was repeated three times with similar results.

We previously screened 96 wild tomato accessions for *Pst*19 resistance using a high-throughput seedling flooding assay (Hassan et al., 2017). In subsequent screens, we identified an additional wild accession, *S. pimpinellifolium* LA1589, with *Pst*19 resistance. LA1589 seedlings displayed resistance to infection (28/34, 82%) whereas susceptible RioGrande-*PtoR* (RG-*PtoR*) seedlings died (0/9, 100%) (**Figure 1A**). To quantitatively confirm our phenotypic observations, we carried out bacterial growth assays on LA1589 (n=23) and Moneymaker-*PtoS* (MM-*PtoS*) (n=10) seedlings flooded with *Pst*19. *Pst*19 grew to log 7 in LA1589 seedlings (n=23) compared to log 8 growth in MM-*PtoS* seedlings (n=10) (**Figure 1B**). Therefore, LA1589 supported 1 log less bacterial growth compared to the susceptible MM-*PtoS* cultivar.

To determine whether resistance to *Pst*19 is maintained in adult plants, we carried out dip inoculations with RG-*PtoR* plants grown to the 4- to 6 leaf stage and assessed the plants for disease symptoms 7 days past infection (dpi). We previously confirmed that MM*-PtoS*, MM*-PtoR*, RG*-PtoS* and RG*-PtoR* are suitable as controls since all are susceptible to *Pst*19 and show similar severe disease symptoms (Hassan et al., 2017). LA1589 exhibited very few specks on leaves and very mild disease symptoms, compared to the control, RG-*PtoR* (**Figure 1C**). RG-*PtoR* displayed numerous lesions and leaf collapse. To quantitatively confirm these phenotypic observations, we measured bacterial growth in LA1589 plants. LA1589 (n=10) supported log 6.5 growth of *Pst*19, approximately 1.3 log less growth than that observed in MM-*PtoS* (n=5, log 7.8) at 5 dpi (**Figure 1D**). Taken together, these qualitative and quantitative results support resistance in LA1589 seedlings and adult plants to *Pst*19.

### *S. pimpinellifolium* LA1589 displays *Pto*-dependent and *Pto*-independent resistance to *Pseudomonas syringae* pv. *tomato*

**Figure 2:**
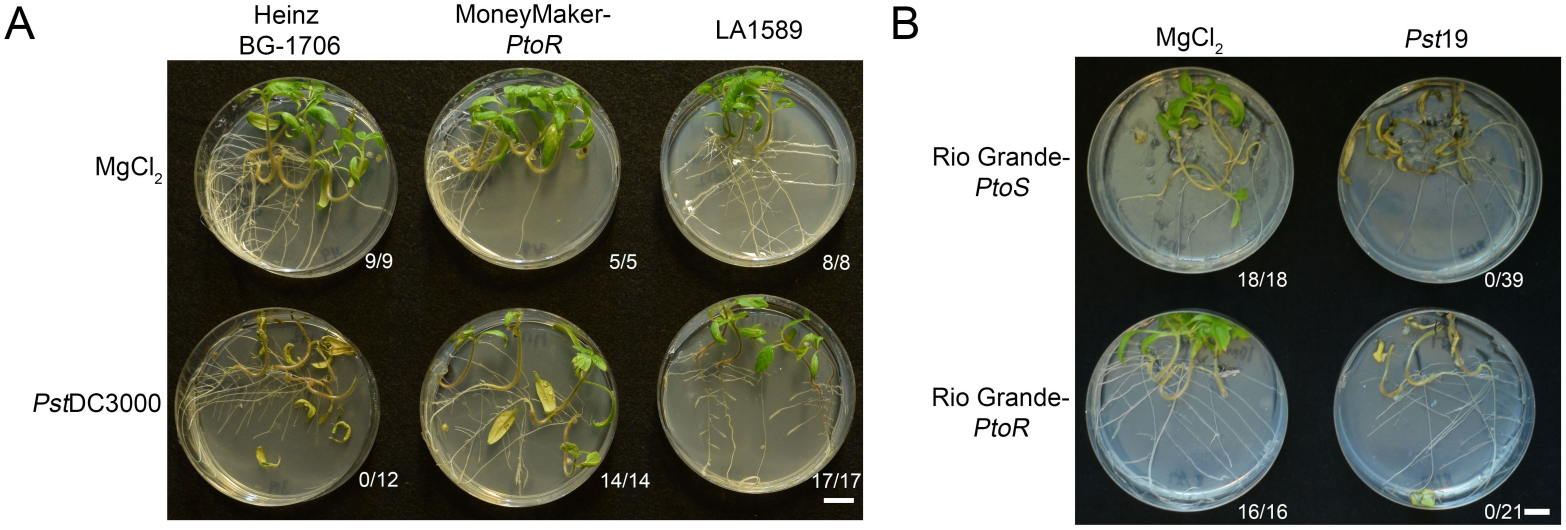
LA1589 seedlings display *Pto*-dependent resistance to *Pst*DC3000. **(A)** Phenotypic resistance or disease symptoms in Heinz BG-1706, MoneyMaker-*PtoR* and LA1589 seedlings 7-10 days post-infection with *Pst*DC3000 at an OD_600_ of 0.005. Heinz BG-1706 seedlings lack the *Pto*/*Prf* gene cluster and are susceptible to *Pst*DC3000. MoneyMaker-*PtoR* and LA1589 seedlings carry the *Pto*/*Prf* gene cluster and are resistant. The number of surviving seedlings out of the total number tested is shown under each plate. The scale bar is 1 cm. **(B)** Rio Grande seedlings with (*PtoR*) and without (*PtoS*) the *Pto/Prf* gene cluster are susceptible to *Pst*19 flooded at an OD_600_ of 0.0075. Disease symptoms in Rio Grande-*PtoS* and Rio Grande-*PtoR* seedlings are shown 7-10 days post-infection. The number of surviving seedlings out of the total number tested is shown under each plate. The scale bar is 1 cm.

*S. pimpinellifolium* was the original source of the *Pto/Prf* cluster (*PtoR*), which confers resistance to race 0 strains (Pitblado and MacNeill, 1983; Pedley and Martin, 2003). To confirm that LA1589 can recognize a race 0 strain, we flood inoculated seedlings with *Pseudomonas syringae pv. tomato* DC3000 (*Pst*DC3000). As expected, all LA1589 seedlings (n=17) and the positive control line, Moneymaker-*PtoR* (MM-*PtoR*) (n=14), were resistant to *Pst*DC3000 (**Figure 2A**). We also tested Heinz BG-1706 which does not carry the *Pto/Prf* gene cluster, and found that all Heinz BG-1706 seedlings (n=12) were susceptible to *Pst*DC3000 (**Figure 2A**). LA1589 carries the *Pto* resistance locus and shows a hypersensitive response to Race 0 strain *Pst*DC3000 in adult plants (Sun et al., 2011). These results are consistent with the Pto/Prf cluster being functional in LA1589 seedlings, allowing the recognition of effectors from *Pst*DC3000.

While *Pst*DC3000 and *Pst*19 both infect tomato, *Pst*19 is closely related to *Pst*T1 (Almeida et al., 2009; Kunkeaw et al., 2010), which is a distinct strain with a different effector complement compared to *Pst*DC3000. *Pst*T1 and *Pst*19 do not carry *avrPto* and do not appear to accumulate AvrPtoB (Lin et al., 2006; Kunkeaw et al., 2010). To determine whether *Pst*19 might express a low level of *avrPtoB* which could be recognized through Pto/Prf in LA1589, we carried out seedling flood assays on RG-*PtoR* (n=21) which contains the *Pto/Prf* locus and RG-*PtoS* (n=39) which lacks the *Pto/Prf* locus. All seedlings died in both genotypes, regardless of whether the lines had or lacked the *Pto/Prf* cluster. This indicates that resistance to *Pst*19 in LA1589 is independent of *Pto/Prf* (**Figure 2B**). This result is consistent with previous work showing that AvrPtoB in *Pst*19 is not recognized by *S. habrochaites* LA2109, even though it carries the *Pto/Prf* cluster (Bao et al., 2015).

### *Pst*19 does not elicit a hypersensitive response characteristic of ETI

**Figure 3:**
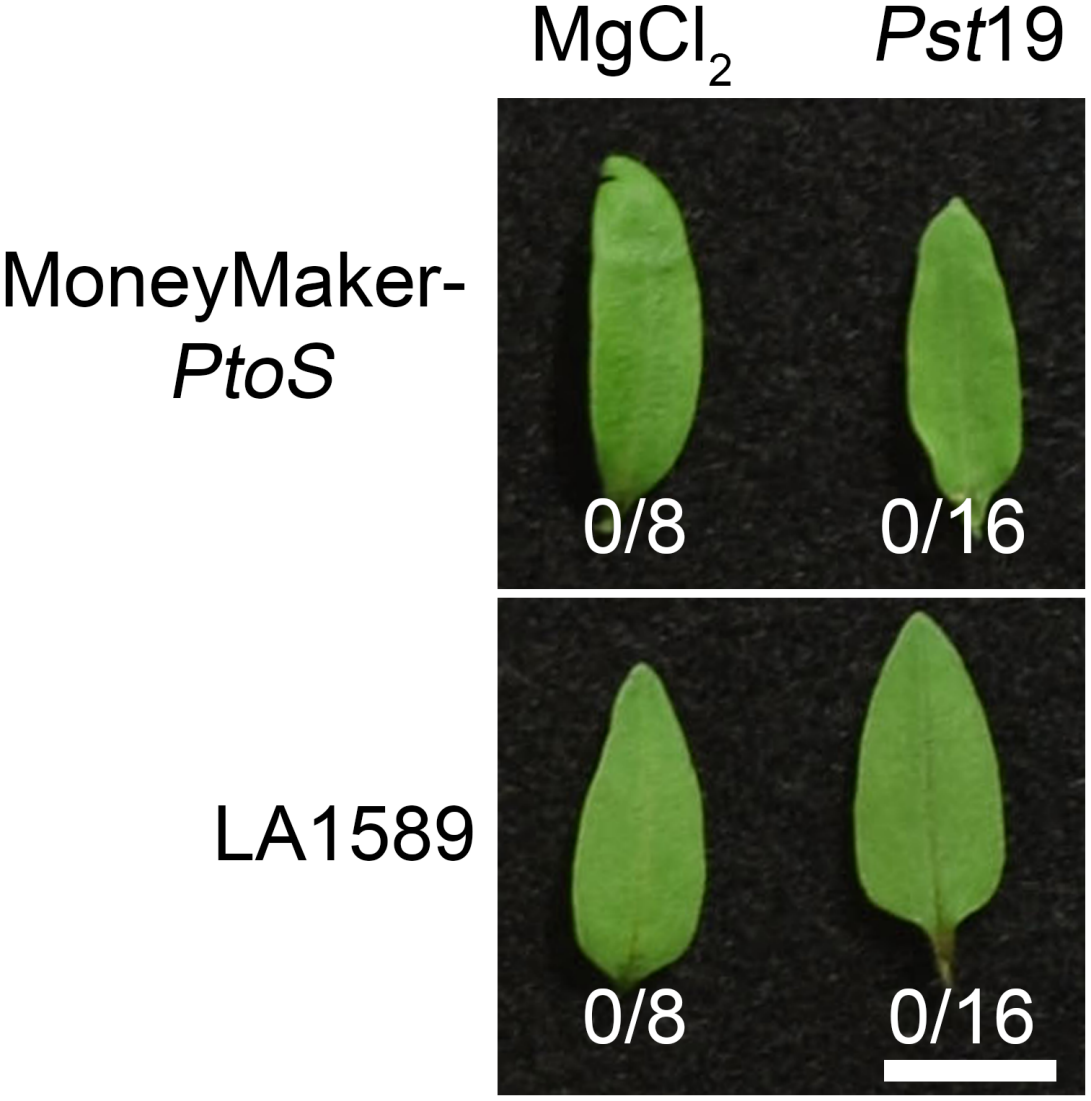
Resistance to *Pst*19 in LA1589 is not associated with a hypersensitive response. 10 day-old Moneymaker-*PtoS* or LA1589 seedlings were pressure infiltrated with ~ 5.0 x 10^7^ cfu/mL *P. syringe* pv. *tomato* 19 (*Pst*19). Plants were photographed 18-22 hours after infiltration. The number of leaves exhibiting an HR is shown under the leaves. The scale bar is 1 cm.

To determine whether recognition of a T3SE in *Pst*19 may cause ETI, we investigated whether *Pst*19 can trigger an HR in LA1589. We infiltrated 10-day old seedlings of LA1589 or MM-*PtoS* (which lacks the *Pto/Prf* locus) with *Pst*19, as previously described (Hassan et al., 2017). *Pst*19 did not trigger an HR in LA1589 (0/16) or MM-*PtoS* seedlings (0/16) (**Figure 3**). These results, taken together with the modest reduction in *Pst*19 growth in LA1589 seedlings (**Figures 1B**, **3**), suggest that resistance in LA1589 is not likely mediated by NLRs.

### QTL mapping using *S. pimpinellifolium* LA1589 inbred backcross lines identifies three genomic regions associated with *Pst*19 resistance

To identify genomic regions associated with *Pst*19 resistance in LA1589, we took advantage of a previously generated inbred backcross line (IBL) population from a cross between *S. lycopersicum* E-6203 and *S. pimpinellifolium* LA1589 (Doganlar et al., 2002). The IBLs were generated after two backcrosses and 6 generations of inbreeding via single seed descent (BC_2_F_6_) and are highly homozygous (Doganlar et al., 2002). Of the original 100 IBLs with uniform genome coverage and map resolution (Doganlar et al., 2002), we were able to obtain 87 lines from the Tomato Genetics Resource Center.

We conducted at least two independent flood inoculation assays for each IBL and screened approximately 20 individuals in total for each of 87 homozygous BC2F6 lines in the seedling assay. For 26 IBLs, at least one seedling survived 10-14 dpi (**Supplementary Table S1**). We identified 4 IBLs with 30-49% seedling survival (Group 1), 9 IBLs with 10-19% survival (Group 2) and 13 IBLs with 1-9% seedling survival (Group 3) (**Supplementary Table S1**). *Pst*19-resistant seedlings displayed a healthy shoot apical meristem and new green vegetative growth. For 61 IBLs, all seedlings were susceptible to *Pst*19 infection (**Supplementary Table S1**). Susceptible seedlings were dead and displayed brown apical meristems and a lack of new growth. The recurrent parent E-6203 consistently died when flooded with *Pst*19 as a susceptible control in the screen (**Supplementary Figure S1**).

**Figure 4:**
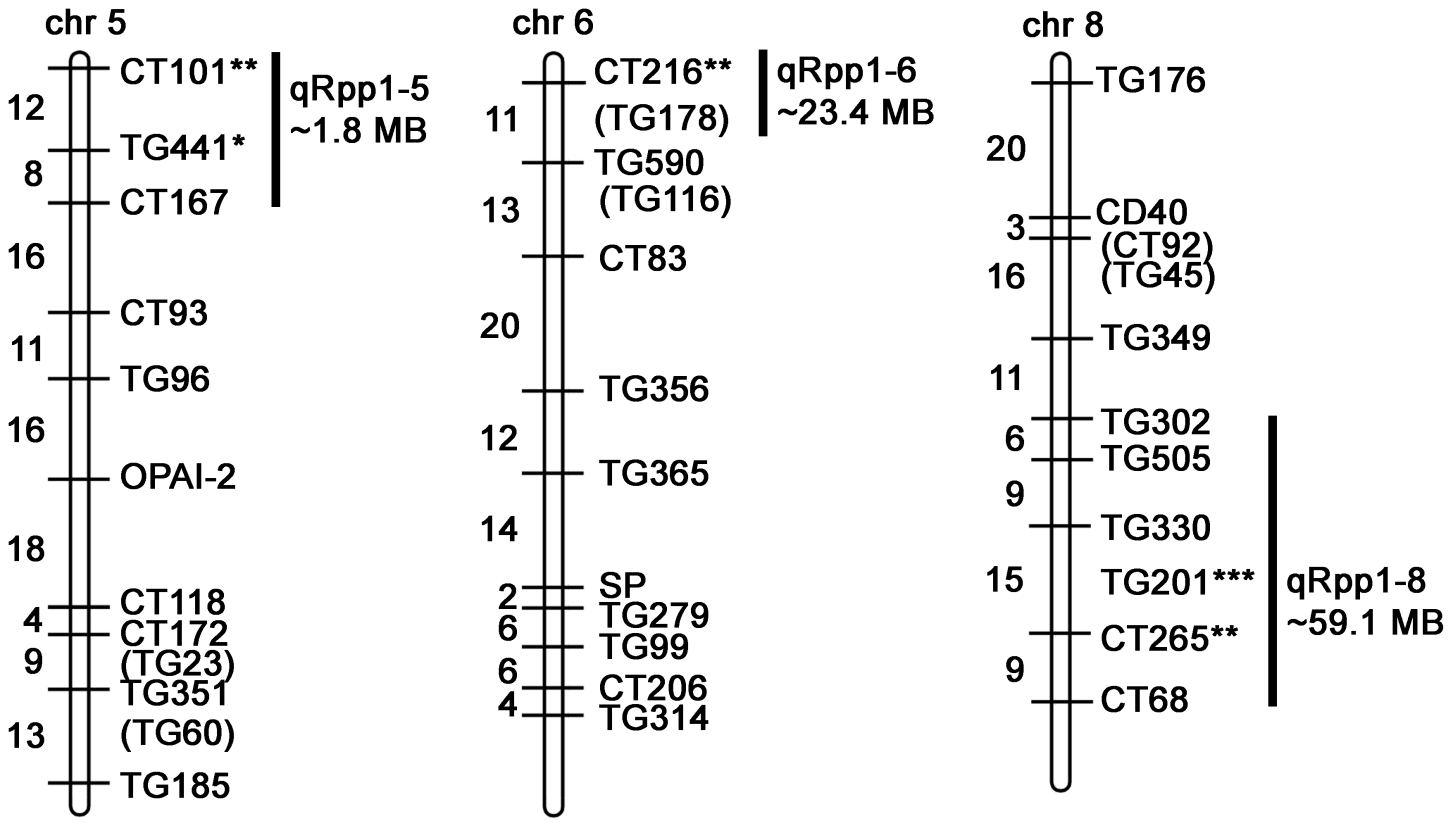
Schematic of markers on chromosomes 5, 6, and 8 in the IBL population indicating QTL associated with resistance (qRpp). Map is modified from (Doganlar et al., 2002) and https://solgenomics.net/cview/map.pl?map_version_id=26. Genetic distances are indicated to the left of the chromosomes (Doganlar et al., 2002). Regions encompassing highly significant markers (*** p<0.01), significant markers (** p<0.05) and/or suggestive markers (* p<0.1) are indicated. Adjacent markers that are not linked with resistance delineate the maximum region of interest (**Supplementary Table S2**). Some marker sequences were not available (**Supplementary Table S3**). The distance between markers was determined based on blasting the Heinz BG-1706 genome with the marker sequences (**Supplementary Table S3**).

To detect genetic associations with *Pst*19 resistance, we undertook single marker analysis based on the frequency of seedling survival and the presence of restriction fragment length polymorphism (RFLP) markers homozygous for LA1589. Three QTL were identified and were named quantitative resistance to *Pst*19 in *S. pimpinellifolium* (qRpp1-5, qRpp1-6, qRpp1-8). Markers with a p-value<0.01 were considered highly significant, markers with a p-value<0.05 were considered significant, markers with a p-value<0.1 were considered suggestive. Suggestive markers were only used to delineate regions when suggestive marker was directly adjacent to a highly significant or significant marker. qRpp1-5 contains one significant marker CT101 (p<0.05), and one suggestive marker TG441 (p<0.1) on chromosome 5 (**Table 1**; **Figure 4**). The next marker on chromosome 5, CT167, was not linked with resistance and was used to delineate the resistance interval of ~1.8 MB (**Figure 4**). qRpp1-6 contains one significant marker CT216 (p<0.05) (**Table 1**; **Figure 4**) on chromosome 6. The next marker on chromosome 6, TG178, was not linked with resistance and the resistance interval is ~23.4 MB. qRpp1-8 has one highly significant marker TG201 (p<0.01) and one significant marker CT265 (p<0.05) at the bottom of chromosome 8 (**Table 1**; **Figure 4**). TG201 had the greatest significance of all the markers. The qRpp1-8 interval on chromosome 8 is ~59.1 MB, and is delineated by two unlinked markers, CT302 and CT68. Based on the consensus genetic linkage map for the IBLs, which was generated with estimated distances between markers, the CT302 marker is quite distant from the markers of interest. CT302 had to be used because TG330 and TG505, which were originally identified as RFLPs from *S. pimpinellifolium*, lacked genotypic information, likely because RFLPs cannot always be easily scored and interpreted.

**Table 1:** Identification of molecular markers linked to *Pst*19 resistance in LA1589 using single marker analysis.

QTL name	Marker	Chromosome #	P-value^1^
qRpp1-5	CT101**	5	0.04
	TG441*	5	0.06
qRpp1-6	CT216**	6	0.03
qRpp1-8	TG201***	8	0.006
	CT265**	8	0.02

^1^Significance of markers determined using Wilcoxon Mann-Whitney test, ***p<0.01 highly significant, **p<0.05 significant, *p<0.1 suggestive.

To refine the genomic intervals associated with resistance, we mapped relevant markers onto the genetic map by blasting the Heinz BG-1706 genome (build 4.0) with RFLP marker sequences available through the SOL Genomics Network (SGN, https://solgenomics.net/). We were able to identify genomic sequences for all significant, suggestive or adjacent markers except TG330, TG505, TG201 and CT265 (all on chromosome 8) which also lacked RFLP sequence information (**Supplementary Tables S2**–**S3**). The intervals identified in the IBL analysis contained 244 genes on chromosome 5, 586 genes on chromosome 6, and 1027 genes on chromosome 8 (**Table 2**; **Figure 4**).

**Table 2:** Number of genes and loci in LA1589 delineated by IBL markers and associated with resistance to *Pst*19.

Chromosome	Delineated markers	# of genes	Loci in LA1589
5	0-CT167	244	Spim05g005010-Spim05g007440
6	0-TG178	586	Spim06g005010-Spim06g010850
8	TG302-CT68	1027	Spim08g020700-Spim08g030960

**Figure 5:**
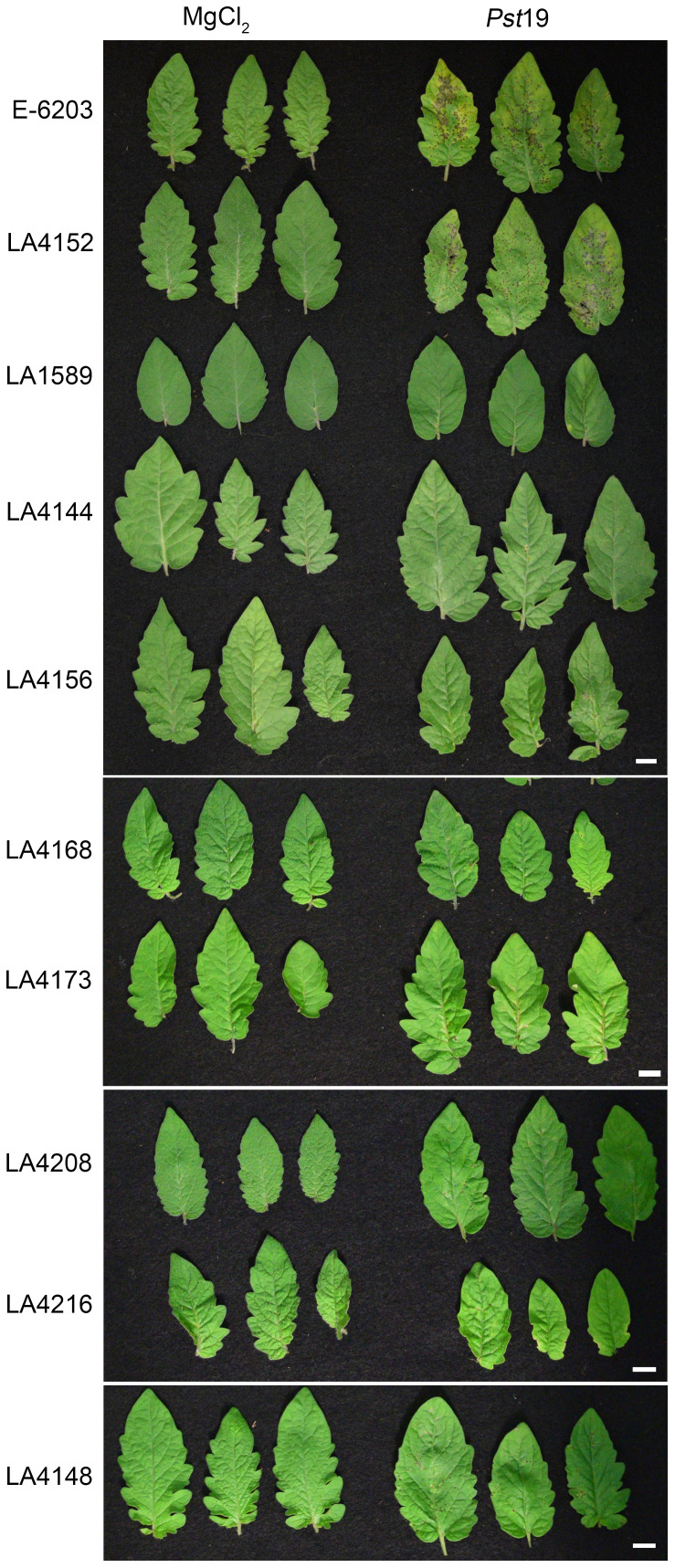
Inbred backcross lines carrying markers associated with *Pst*19 resistance show weaker disease symptoms during *P. syringae* pv. *tomato* 19 (*Pst*19) infection than a susceptible cultivar. Susceptible cultivar E-6203, susceptible IBL LA4152, resistant LA1589 and seven IBLs carrying markers associated with resistance (LA4144, LA4156, LA4168, LA4173, LA4208, LA4216 and LA4148) were dip-inoculated with a bacterial suspension at an OD_600_ of 0.2 at the 4-6-week-old adult stage. Leaves were photographed 6-7 days after infection. The scale bar indicates 1 cm.

Based on the single-marker analysis that identified highly significant, significant or suggestive markers from LA1589 associated with *Pst*19 resistance in seedlings, we selected IBLs with various combinations of these markers (**Table 1**; **Supplementary Table S4**) to test for resistance in adult plants. Previous analysis of the IBLs had determined whether markers were homozygous for LA1589, homozygous for E-6203, or heterozygous with both LA1589 and E-6203 (**Supplementary Table S4**; **Figure 5**) (Doganlar et al., 2002). No IBLs were homozygous for LA1589 at all four highly significant or significant markers associated with the QTL. We prioritized testing seven IBLs as adult plants because they showed higher levels of resistance as seedlings (**Supplementary Table S1**), 4 from Group 1 with 30-49% resistance (LA4156, LA4168, LA4208, LA4216) and 3 from Group 2 with 10-19% resistance (LA4144, LA4148, LA4173). We dip-inoculated adult plants from LA1589, the seven IBLs with higher levels of resistance, a susceptible IBL (LA4152), and the susceptible E-6203 cultivar with *Pst*19, and assessed the development of disease symptoms. Typical symptoms of bacterial speck include brown or dark brown necrotic lesions surrounded by chlorotic halos. All seven of the resistant IBLs (LA4144, LA4148, LA4156, LA4168, LA4173, LA4208, LA4216) displayed reduced lesions, compared to the susceptible lines E-6203 and LA4152. However, the IBLs displayed more lesions than LA1589, which had the fewest lesions (**Figure 5**).

**Figure 6:**
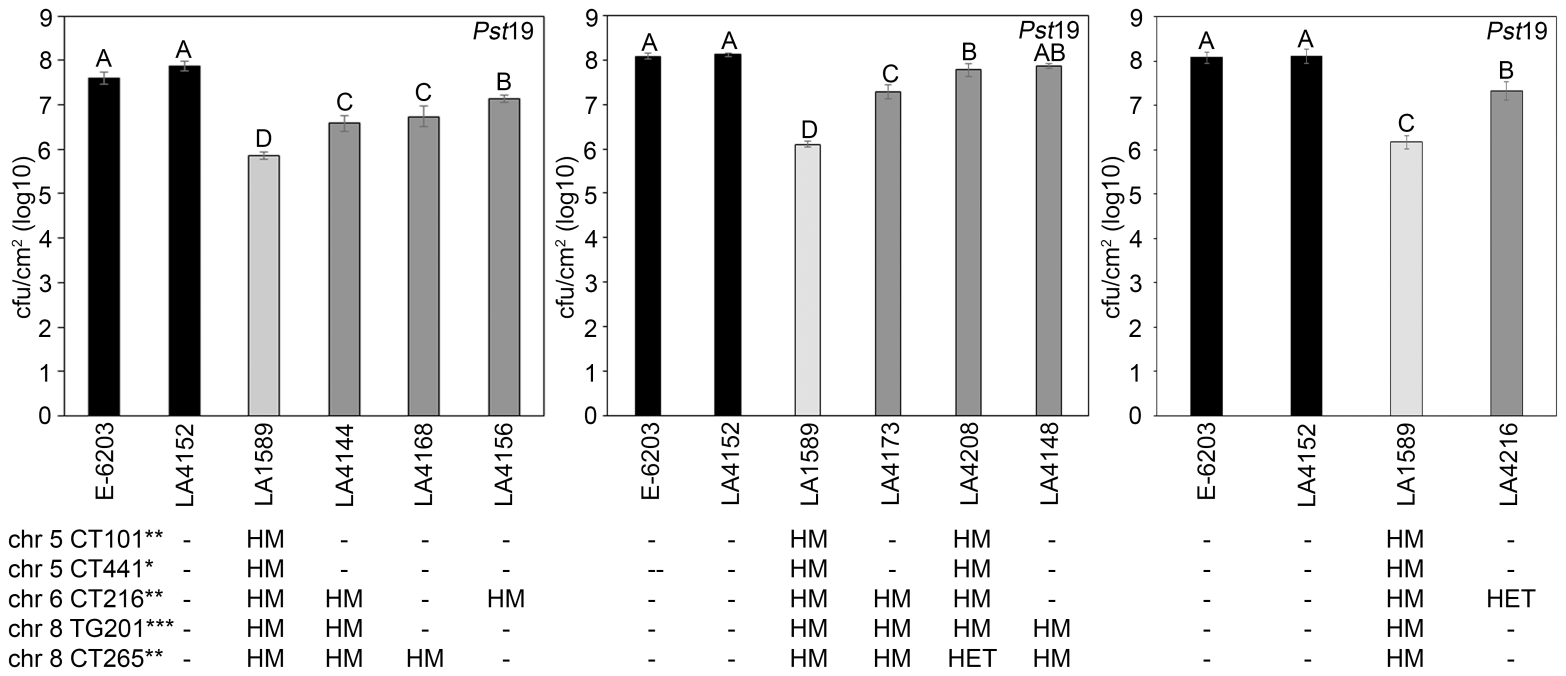
Inbred backcross lines carrying markers associated with *Pst*19 resistance support less growth of *P. syringae* pv. *tomato* 19 (*Pst*19). Susceptible cultivar E-6203 (black bar), susceptible IBL LA4152 (black bar), resistant LA1589 (light grey bar) and seven IBLs carrying markers associated with resistance LA4144, LA4148, LA4156, LA4168, LA4173, LA4208 and LA4216 (dark grey bars) were dip-inoculated with a bacterial suspension at an OD_600_ of 0.2 at the 4-6-week-old adult stage. Bacterial counts and log growth were determined at 5 days past infection (dpi) in E-6203, LA4152, LA1589, LA4144, LA4168 and LA4156 (left panel), E-6203, LA4152, LA1589, LA4173, LA4208 and LA4148 (middle panel), and E-6203, LA4152, LA1589 and LA4216 (right panel). Within each experiment, significant differences among genotypes are shown with different letters. One-factor ANOVA using a GLM procedure (p<0.05) followed by the Tukey’s *post hoc* test, a multiple comparison of means test, was used to determine statistical differences. The error bars indicate standard error. Experiments for each IBL were repeated three times with similar results. The genotypes of the IBLs are shown below the growth assay with the chromosome location and marker genotype for each highly significant (***p<0.01), significant (**0.01<p<0.05), or suggestive (*0.05<p<0.1) marker (see also **Table 1**; **Supplementary Table S4**). HM indicates homozygous LA1589. HET indicates heterozygous LA1589/E-6203. - indicates homozygous E-6203. The genotype of LA1589 is inferred. qRpp1-5 includes CT101 and TG441. qRpp1-6 includes CT216. qRpp1-8 includes TG201 and CT265.

To quantitatively confirm these phenotypic results, we measured bacterial growth in all seven IBLs and compared them to the susceptible E-6203 cultivar and susceptible IBL LA4152, as well as the resistant accession LA1589. Five IBLs, LA4144 (log 6.6), LA4156 (log 7.1), LA4168 (log 6.7), LA4173 (log 7.3) and LA4216 (log 7.3) displayed between an 8-to-10-fold reduction in bacterial growth, compared to the parental line, E-6203 and a 7-to-13-fold reduction, compared to the susceptible IBL LA4152 (**Figure 6**). One IBL, LA4208 (log 7.8) displayed a more modest but still significant 3-fold reduction in growth, compared to E-6203 and LA4152 (**Figure 6**). LA4148 supported similar levels of bacterial growth as LA4208 but these differences were not statistically significant compared to E-6203 and LA4152 (**Figure 6**). Bacterial growth in all IBLs was, however, significantly higher than in LA1589, ranging from 0.7-1.9 log higher across these lines. These results are consistent with the intermediate symptoms observed in these lines as compared to LA1589 (**Figure 5**). The combination of LA1589 markers within the IBLs contributed to a partial reduction in bacterial growth, but did not recapitulate the complete reduction in bacterial growth observed in LA1589.

To further confirm that LA1589 resistance to *Pst*19 is Pto/Prf-independent, we analyzed our IBL data for associations with *Pto/Prf*. The *Pto/Prf* locus is located around the middle of chromosome 5 (**Supplementary Table S5**). The delineated regions from the IBL analysis are at the tip of chromosome 5 and do not overlap (**Table 1**; **Figure 4**). This is consistent with our finding that *Pst*19 is not recognized by Pto/Prf (**Figure 2B**) and previous findings that AvrPtoB in *Pst*19 is not recognized (Bao et al., 2015). We also examined whether the delineated regions include *FLS2.1*, *FLS2.2* or *FLS3* since LA1589 has been previously shown to be responsive to flgII-28 from *Pst*19 (Hind et al., 2016). *FLS2.1* and *FLS2.2* are found on chromosome 2 and *FLS3* is found on chromosome 4 (**Supplementary Table S5**). We did not find any associations between these two chromosomes and the resistance phenotype (**Table 1**; **Figure 4**).

### F2 mapping population approach identifies candidate genes for resistance

To refine the genetic intervals from the IBLs and identify SNPs associated with resistance, we generated an F2 mapping population from a cross between LA1589 and the *Pst*19-susceptible cultivar Heinz BG-1706 (**Supplementary Figure S1**). We infected and phenotyped 1181 plants for resistance or susceptibility to *Pst*19, using the previously described criteria. We found that 18% (n=215) were resistant and 82% (n=966) were susceptible (**Table 3**). The segregation ratios are not Mendelian, which is consistent with the IBL data showing that multiple loci contribute to resistance (**Table 1**). To further refine the resistance or susceptibility phenotypes, we categorized seedlings as strongly resistant (6.7%), moderately resistant (3.7%), weakly resistant (7.8%), susceptible (62%) and highly susceptible (20%) (**Table 1**). Strongly resistant seedlings displayed the greatest degree of branching and new growth compared to other seedlings at 14 dpi. Moderately resistant seedlings had some branching and new growth, and weakly resistant seedlings had very little branching and one or two newly emerging leaves. Highly susceptible seedlings had brown apical meristems and no new green growth at 7-9 dpi, whereas susceptible seedlings took longer to exhibit these phenotypes. To maximize the identification of potential genetic differences contributing to the resistance phenotype, we selected individuals at the phenotypic extremes: 79/1181 (6.7%) seedlings with strong resistance, and 233/1181 (20%) with high susceptibility. We extracted DNA from a pool of 79 seedlings with strong resistance, and a pool of 233 highly susceptible individuals, and carried out whole-genome sequencing. We obtained 348-397X coverage of the genome, where 73-74% of reads for the strongly resistant pool and 65-67% of reads for the highly susceptible pool had a BWA MEM quality alignment score of 20 or higher (**Supplementary Table S6**).

**Table 3:** *Pst*19 resistance in F2 segregating population of Heinz BG-1706 x LA1589 seedlings.

Phenotype	Number of plants (percentage)	Subcategory	Number of plants (percentage)
Susceptible	966 (82%)	Highly susceptible	233 (20%)
		Susceptible	733 (62%)
Resistant	215 (18%)	Strongly resistant	79 (6.7%)
		Moderately resistant	44 (3.7%)
		Weakly resistant	92 (7.8%)
Total	1181		1181

Mendelian 3:1 segregation ratio rejected (Chi-squared test p<0.001).

**Figure 7:**
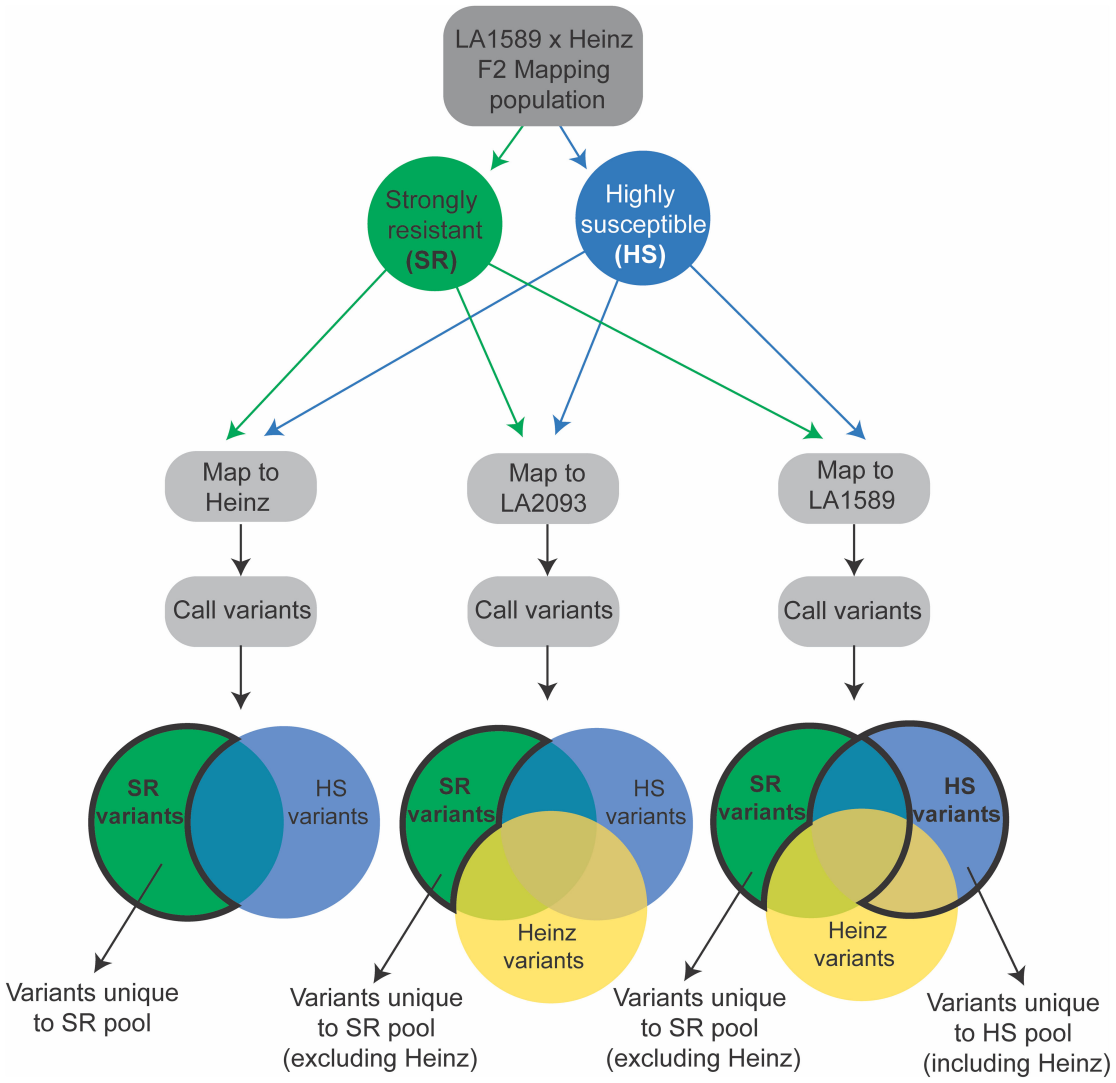
Schematic of variant calling pipeline to susceptible Heinz BG-1706, susceptible *S. pimpinellifolium* LA2093 and resistant *S. pimpinellifolium* LA1589. SR indicates strongly resistant (green) and HS indicates highly susceptible (blue). Heinz variants are shown in yellow. Variants of interest in the Venn diagrams are shown with a bold black outline.

We separately mapped reads from the strongly resistant or highly susceptible pools to the Heinz BG-1706, *S. pimpinellifolium* LA2093 and *S. pimpinellifolium* LA1589 reference genomes (Sato et al., 2012; Alonge et al., 2020; Wang et al., 2020) and identified variants (**Figure 7**). We included *S. pimpinellifolium* LA2093, as it is susceptible to *Pst*19 (**Supplementary Figure S1**). For reads mapped to susceptible Heinz BG-1706, we identified unique and over-represented variants in the LA1589 highly resistant pool, as these were most likely to be associated with resistance in LA1589 (**Figure 7**, left, outlined in bold black). To eliminate variants commonly found in *S. pimpinellifolium* that do not contribute to resistance, we mapped reads to the susceptible *S. pimpinellifolium* LA2093 genome and looked for variants unique to the strongly resistant pool (**Figure 7**; **Supplementary Figure S1**). Since variants in the strongly resistant pool mapped to LA2093 may come from the cross with Heinz BG-1706, we removed variants specific to Heinz BG-1706. This enabled us to identify unique variants specific to LA1589 which may contribute to resistance (**Figure 7**, middle, outlined in bold black). To identify LA1589 orthologs to Heinz BG-1706 or LA2093 genes, we used the Reciprocal Best Hits method (Ward and Moreno-Hagelsieb, 2014). If no orthologs were identified, the closest homologs were identified using BLAST based on low bit-scores and low e-values (Pearson, 2013). Lastly, for reads mapped to *S. pimpinellifolium* LA1589, unique SNPs in the highly susceptible pool could come from either parent and might disrupt resistance. These SNPs are of interest if they differ between the strongly resistant and highly susceptible pools (**Figure 7**, right, blue bubble outlined in bold black). We also assumed that a small number of SNPs could be unique to the strongly resistant pool since there could be some diversity between the sequenced LA1589 and the TGRC stock of LA1589 (**Figure 7**, right, green bubble outlined in bold black). Variants relative to LA1589 that are present in Heinz BG-1706 and the strongly resistant pool are not useful and were eliminated. Based on all three approaches combined with the QTL from the IBLs, we identified 5 candidate genes, with unique variants in the resistant pool compared to the susceptible pool. qRpp1-6 contains one candidate gene: a SUMO-domain containing protein. qRpp1-8 contains four candidate genes: a protein phosphatase 2C (PP2C), a pectin acetylesterase, a copper chaperone for superoxide dismutase, and ribosomal protein L16. No candidate genes were identified for qRpp1-5.

## Discussion

Genetic resistance is extremely effective in protecting plants from infection. It has been very challenging to identify sources of resistance in wild populations and to have sufficient resolution to identify genes associated with resistance. Here, using our high-throughput seedling flood assay, we identified resistance to *Pst*19 in the wild tomato species *S. pimpinellifolium* LA1589 (**Figure 1**). Interestingly, although *S. pimpinellifolium* was the original source of the *Pto/Prf* gene cluster (Pilowsky and Zutra, 1982; Pitblado and MacNeill, 1983; Pedley and Martin, 2003), resistance to *Pst*19 in LA1589 is independent of *Pto/Prf*, and does not result in an HR (**Figures 2**, **3**; **Supplementary Table S5**). By screening an existing set of IBLs, we were able to narrow the genomic regions of interest to ~250 genes on chromosome 5, ~590 genes on chromosome 6 and ~1025 genes on chromosomes 8, representing 3 QTL associated with resistance (**Tables 1**, **2**; **Figure 4**). We generated an independent F2 mapping population between Heinz BG-1706 and LA1589 and carried out next-generation sequencing of pools of seedlings with phenotypes at the extremes of resistance or susceptibility. Qualitatively, highly resistant F2 individuals showed similar resistance as highly resistant LA1589 individuals, and highly susceptible F2 individuals showed similar susceptibility as Heinz BG-1706. We analyzed variants to identify those that were specific to the resistant pool and missing from the susceptible pool or the susceptible parent cultivar. This analysis enabled us to shortlist five candidates as having unique variants in the resistant pool (**Table 4**).

**Table 4:** Candidate genes associated with resistance from IBL and GBS analysis.

QTL	Gene number^1^	Annotation	Identified by mapping to:
qRpp1-6	SPIMP06g0183860	SUMO-domain containing	LA2093
qRpp1-8	Spimp08g024080	Protein phosphatase 2C	LA1589
qRpp1-8	SPIMP08g0250550	Pectin acetylesterase	LA2093
qRpp1-8	Solyc08g079830	Copper chaperone for superoxide dismutase	Heinz BG-1706
qRpp1-8	Solyc05g045840	Ribosomal protein L16	Heinz BG-1706

^1^Gene numbers are in reference to the genome used in mapping, as shown in last column.

No candidate genes were identified for qRpp1-5.

Further characterization of resistance in *S. pimpinellifolium* LA1589 suggests that NLR-mediated ETI is unlikely to contribute to the resistance to *Pst*19. LA1589 seedlings supported about 1 log less bacterial growth than MM-*PtoS*, and LA1589 adults supported about 1.3 log less bacterial growth than MM-*PtoS* (**Figures 1B, D**). This difference is much smaller than typical 2-3 log difference observed when ETI is involved (Hassan et al., 2017). Importantly, we see similar resistance in both seedlings and adults, as we have previously observed in other accessions (Hassan et al., 2017). In addition, we did not observe an HR (**Figure 3**), which is commonly observed with ETI. While *S. pimpinellifolium* LA1589 carries the *Pto/Prf* locus that recognizes AvrPto and AvrPtoB (**Figure 2A**, it is not sufficient to confer resistance to *Pst*19 (**Figure 2B**). Consistent with this, the *Pto/Prf* locus is not located in the regions identified for *Pst*19 resistance in the IBLs (**Tables 1**, **2**; **Figure 4**; **Supplementary Table S5**). In addition, previous work showed that AvrPtoB from *Pst*19 is not recognized in *S. habrochaites* (Bao et al., 2015), further supporting that the observed resistance is independent of Pto/Prf. We also confirmed that *FLS2.1*, *FLS2.2* and *FLS3* are not present in any of the delineated regions (**Tables 1**, **2**; **Figure 4**; **Supplementary Table S5**). These data further support the presence of additional sources of *Pst*19 resistance in *S. pimpinellifolium* LA1589. Since *S. pimpinellifolium* was introgressed into many early tomato cultivars (Pilowsky and Zutra, 1982; Pitblado and MacNeill, 1983; Pedley and Martin, 2003), some resistance to *Pst*19 might already exist in breeding materials (Menda et al., 2014), which would expedite the introduction of new sources of resistance.

Our IBL screen identified specific chromosomal regions that were associated with reduced symptoms and lower bacterial growth (**Table 2**; **Figures 4**–**6**). These data demonstrate that multiple genes contribute to resistance to *Pst*19 (**Figures 4**–**6**), which is consistent with QDR. No IBLs were available that were homozygous for all resistance QTL identified here. IBLs that carry some but not all linked markers show more disease and smaller reductions in bacterial growth compared to the LA1589 parent (**Figures 5**, **6**). It is important to note that the IBLs have only been genotyped at specific markers. Thus, even though different IBLs may carry the same homozygous LA1589 markers, they may contain different flanking genomic regions from *S. pimpinellifolium*. IBLs lacking all linked resistance markers (ie. LA4152) were as susceptible as the cultivar E-6203, with severe disease symptoms and high levels of bacterial growth (**Figures 5**, **6**). This supports the contribution of the QTL to disease resistance. Interestingly, *S. pimpinellifolium* LA1589 was previously identified as having resistance to the *P. syringae* race 1 strain A9 (Thapa et al., 2015). However, the observed resistance to *Pst*A9 was much weaker than the resistance we observed against *Pst*19 (**Figure 1**), and was not pursued. QDR is a powerful tool in protecting plants from infection, as it typically involves many genes of small to moderate effect. As a result, resistance mediated by QTL is typically harder for pathogens to overcome (Poland et al., 2009; French et al., 2016). QDR can involve genes with predicted roles in immunity, such as receptor-like kinases and NLRs, as well as genes with different molecular functions (Bao et al., 2015; Debieu et al., 2016; Gonzalez et al., 2017; Guo et al., 2020). Some QTL are associated with effector recognition, such as those involved in the recognition of HopAM1 (Iakovidis et al., 2016) or HopQ1-1 (Luo et al., 2017).

To gain greater resolution into the genes involved in resistance to *Pst*19 within the intervals defined by the IBL analysis, we coupled the IBL screen with a genotyping-by-sequencing screen on a separate F2 mapping population derived from *S. pimpinellifolium* LA1589, and Heinz, which is susceptible to *Pst*19 (**Supplementary Figure S1**). High-quality genome sequences are available for both Heinz and *S. pimpinellifolium* LA1589 (Sato et al., 2012; Wang et al., 2020; Takei et al., 2021). We screened ~1200 individuals for their resistance and susceptibility phenotypes, which did not segregate in a Mendelian manner (**Table 3**), consistent with the IBL analysis. This population is likely to have more recombination events than the IBLs, which can help identify genes of interest. By focusing on the regions identified by the IBLs and using custom bioinformatic pipelines, we were able to identify unique SNPs that were specific to the resistant pool of the F2 mapping population, but not present in Heinz, the susceptible pool of the F2 mapping population or susceptible *S. pimpinellifolium* accession LA2093 (**Figure 7**, **Supplementary Table S1**). For SNPs in Heinz genes, we identified *S. pimpinellifolium* homologs with high similarity by reciprocal best hit Blast. The *S. pimpinellifolium* genes in the strongly resistant pool had unique differences compared to Heinz or the highly susceptible pool.

Some of our candidate genes have predicted functions that have been shown to be involved in resistance (**Table 4**). qRpp1-6 contains one candidate gene: a SUMO-domain containing protein. Sumoylation contributes to disease progression and virulence activities of phytopathogenic bacteria, as well as immune responses against pathogens (Park et al., 2011). Mutants in Arabidopsis the SUMO E3 ligase, SIZ1, or SUMO genes, SUM1/2, have high levels of salicylic acid and constitutive expression of pathogenesis-related (PR genes) (Lee et al., 2007; van den Burg et al., 2010). qRpp1-8 contains four candidate genes: a protein phosphatase 2C (PP2C), a pectin acetylesterase, a copper chaperone for superoxide dismutase, and ribosomal protein L16. Some PP2Cs can regulate PTI and ETI responses to *P. syringae* (Widjaja et al., 2010; Giska and Martin, 2019; Sobol et al., 2022). A citrus pectin acetylesterase was found to be a negative regulator of defenses against *Xanthomonas citri* pv. *citri* (Li et al., 2020). Copper chaperone for superoxide dismutase proteins have been implicated in resistance against *Magnaporthe orzyae*, which causes rice blast, and in production of reactive oxygen species, which is associated with immune responses (Li et al., 2019). Ribosomal proteins have been associated with resistance to different pathogens, including non-host strains of *Pseudomonas syringae* in *Nicotiana* species and virulent strains of *Pseudomonas syringe* in Arabidopsis (Ramu et al., 2020; Son and Park, 2023). It is also possible that some of these genes are susceptibility factors which are targeted by the pathogen for enhanced virulence (Dangl et al., 2013; Schreiber et al., 2021a; Schreiber and Lewis, 2021). When susceptibility factors are disrupted, they can enhance plant resistance (Dangl et al., 2013; Schreiber et al., 2021b; Schreiber and Lewis, 2021).

A previous screen identified QTL on chromosomes 2 and 8 in *S. habrochaites* that contributed to the resistance against *Pst*T1 (Bao et al., 2015), which is very similar to *Pst*19 (Kunkeaw et al., 2010). The QTL on chromosome 8 was not followed up on, however the QTL on chromosome 2 was narrowed to ~140 genes and one candidate gene is a receptor-like protein kinase (Bao et al., 2015). This RLK is distinct from the candidate genes we identified. Another study identified 3 QTL on chromosomes 1, 2 and 12 in *S. habrochaites* that contributed to resistance against a different race 1 strain, *Pst*A9 (Thapa et al., 2015). No candidate genes were identified for these QTL.

The identification of new sources of disease resistance can help bolster plant resilience to infection. Combining classical genetics with next-generation sequencing and high-throughput seedling assays allowed us to identify genomic regions and candidate genes associated with resistance in wild tomato. QTL-seq approaches can expedite the identification of variants associated with phenotypes of interest in many agriculturally relevant crops (Takagi et al., 2013; Singh et al., 2022). Our data may be helpful for plant breeders in prioritizing loci for introgression and/or for replacement in susceptible varieties.

## Data Availability

The datasets presented in this study can be found in online repositories. The names of the repository/repositories and accession number(s) can be found below: https://www.ncbi.nlm.nih.gov/, PRJNA1022300.

## References

[B1] Almeida N. F. Yan S. Lindeberg M. Studholme D. J. Schneider D. J. Condon B.. (2009). A draft genome sequence of *Pseudomonas syringae* pv. *tomato* T1 reveals a type III effector repertoire significantly divergent from that of *Pseudomonas syringae* pv. *tomato* DC3000. Mol. Plant-Microbe Interact. 22, 52–62. doi: 10.1094/MPMI-22-1-0052 19061402

[B2] Alonge M. Wang X. G. Benoit M. Soyk S. Pereira L. Zhang L.. (2020). Major impacts of widespread structural variation on gene expression and crop improvement in tomato. Cell 182, 145. doi: 10.1016/j.cell.2020.05.021 32553272 PMC7354227

[B3] Andrews S. (2010). FastQC: a quality control tool for high throughput sequence data. Available online at: http://www.bioinformatics.babraham.ac.uk/projects/fastqc.

[B4] Bao Z. L. Meng F. H. Strickler S. R. Dunham D. M. Munkvold K. R. Martin G. B. (2015). Identification of a candidate gene in *Solanum habrochaites* for resistance to a race 1 strain of *Pseudomonas syringae* pv. *tomato* . Plant Genome 8. doi: 10.3835/plantgenome2015.02.0006 33228271

[B5] Block A. Alfano J. R. (2011). Plant targets for *Pseudomonas syringae* type III effectors: virulence targets or guarded decoys. Curr. Opin. Microbiol. 14, 39–46. doi: 10.1016/j.mib.2010.12.011 21227738 PMC3040236

[B6] Boyd L. A. Ridout C. O'Sullivan D. M. Leach J. E. Leung H. (2013). ) Plant-pathogen interactions: disease resistance in modern agriculture. Trends Genet. 29, 233–240. doi: 10.1016/j.tig.2012.10.011 23153595

[B7] Cai R. Lewis J. Yan S. Liu H. Clarke C. R. Campanile F.. (2011). The plant pathogen *Pseudomonas syringae* pv. *tomato* is genetically monomorphic and under strong selection to evade tomato immunity. PloS Pathog. 7, e1002130. doi: 10.1371/journal.ppat.1002130 21901088 PMC3161960

[B8] Cingolani P. Patel V. M. Coon M. Nguyen T. Land S. J. Ruden D. M.. (2012a). Using *Drosophila melanogaster* as a model for genotoxic chemical mutational studies with a new program, SnpSift. Front. Genet. 3, 1–9. doi: 10.3389/fgene.2012.00035 22435069 PMC3304048

[B9] Cingolani P. Platts A. Wang L. L. Coon M. Nguyen T. Wang L.. (2012b). A program for annotating and predicting the effects of single nucleotide polymorphisms, SnpEff: SNPs in the genome of *Drosophila melanogaster* strain w(1118); iso-2; iso-3. Fly 6, 80–92. doi: 10.4161/fly.19695 22728672 PMC3679285

[B10] Couto D. Zipfel C. (2016). Regulation of pattern recognition receptor signaling in plants. Nat. Rev. Immunol. 16, 537–552. doi: 10.1038/nri.2016.77 27477127

[B11] Dangl J. L. Horvath D. M. Staskawicz B. J. (2013). Pivoting the plant immune system from dissection to deployment. Science 341, 746–751. doi: 10.1126/science.1236011 23950531 PMC3869199

[B12] Debieu M. Huard-Chauveau C. Genissel A. Roux F. Roby D.. (2016). Quantitative disease resistance to the bacterial pathogen *Xanthomonas campestris* involves an Arabidopsis immune receptor pair and a gene of unknown function. Mol. Plant Pathol. 17, 510–520. doi: 10.1111/mpp.12298 26212639 PMC6638543

[B13] DeFalco T. A. Zipfel C. (2021). Molecular mechanisms of early plant pattern-triggered immune signaling. Mol. Cell 81, 3449–3467. doi: 10.1016/j.molcel.2021.07.029 34403694

[B14] DeYoung B. J. Innes R. W. (2006). Plant NBS-LRR proteins in pathogen sensing and host defense. Nat. Immunol. 7, 1243–1249. doi: 10.1038/ni1410 17110940 PMC1973153

[B15] Doganlar S. Frary A. Ku H. M. Tanksley S. D. (2002). Mapping quantitative trait loci in inbred backcross lines of *Lycopersicon pimpinellifolium* (LA1589). Genome 45, 1189–1202. doi: 10.1139/g02-091 12502266

[B16] French E. Kim B. S. Iyer-Pascuzzi A. S. (2016). Mechanisms of quantitative disease resistance in plants. Semin. Cell Dev. Biol. 56, 201–208. doi: 10.1016/j.semcdb.2016.05.015 27212254

[B17] Giska F. Martin G. B. (2019). PP2C phosphatase Pic1 negatively regulates the phosphorylation status of Pti1b kinase, a regulator of flagellin-triggered immunity in tomato. Biochem. J. 476, 1621–1635. doi: 10.1042/BCJ20190299 31097490

[B18] Gonzalez A. M. Godoy L. Santalla M. (2017). Dissection of resistance genes to *Pseudomonas syringae* pv. *phaseolicola* in UI3 common bean cultivar. Int. J. Mol. Sci. 18. doi: 10.3390/ijms18122503 PMC5751106 29168746

[B19] Guo Z. F. Zou C. Liu X. G. Wang S. H. Li W. X. Jeffers D.. (2020). Complex genetic system involved in *Fusarium* ear rot resistance in maize as revealed by GWAS, bulked sample analysis, and genomic prediction. Plant Dis. 104, 1725–1735. doi: 10.1094/PDIS-07-19-1552-RE 32320373

[B20] Hassan J. A. Zhou Y. J. Lewis J. D. (2017). A rapid seedling resistance assay identifies wild tomato lines that are resistant to *Pseudomonas syringae* pv. tomato race 1. Mol. Plant-Microbe Interact. 30, 701–709. doi: 10.1094/MPMI-11-16-0247-R 28517960

[B21] Hassan J. A. Chau-Ly I. J. Lewis J. D. (2020). High-throughput identification of resistance to *Pseudomonas syringae* pv. *tomato* in tomato using seedling flood assay. Jove-Journal Visualized Experiments 157, 12. doi: 10.3791/60805 32225144

[B22] Heath M. C. (2000). Hypersensitive response-related death. Plant Mol. Biol. 44, 321–334. doi: 10.1023/A:1026592509060 11199391

[B23] Hirano S. S. Upper C. D. (2000). Bacteria in the leaf ecosystem with emphasis on *Pseudomonas syringae* - a pathogen, ice nucleus, and epiphyte. Microbiol. Mol. Biol. Rev. 64, 624–653. doi: 10.1128/MMBR.64.3.624-653.2000 10974129 PMC99007

[B24] Iakovidis M. Teixeira P. Exposito-Alonso M. Cowper M. G. Law T. F. Liu Q. L.. (2016). Effector-triggered immune response in *Arabidopsis thaliana* is a quantitative trait. Genetics 204, 337. doi: 10.1534/genetics.116.190678 27412712 PMC5012398

[B25] Jones J. D. G. Vance R. E. Dangl J. L. (2016). Intracellular innate immune surveillance devices in plants and animals. Science 354, 1117. doi: 10.1126/science.aaf6395 27934708

[B26] Kerr E. A. Cook F. I. (1983). Ontario-7710 - A tomato breeding line with resistance to bacterial speck, *Pseudomonas syringae* pv. tomato (Okabe). Can. J. Plant Sci. 63, 1107–1109. doi: 10.4141/cjps83-146

[B27] Kunkeaw S. Tan S. Coaker G. (2010). Molecular and evolutionary analyses of *Pseudomonas syringae* pv. tomato race 1. Mol. Plant-Microbe Interact. 23, 415–424. doi: 10.1094/MPMI-23-4-0415 20192829

[B28] Lee J. Nam J. Park H. C. Na G. Miura K. Jin J. B.. (2007). Salicylic acid-mediated innate immunity in Arabidopsis is regulated by SIZ1 SUMO E3 ligase. Plant J. 49, 79–90. doi: 10.1111/j.1365-313X.2006.02947.x 17163880

[B29] Lewis J. D. Desveaux D. Guttman D. S. (2009). The targeting of plant cellular systems by injected type III effector proteins. Semin. Cell Dev. Biol. 20, 1055–1063. doi: 10.1016/j.semcdb.2009.06.003 19540926

[B30] Li H. (2013). Aligning sequence reads, clone sequences and assembly contigs with BWA-MEM. arXiv, 1–3. doi: 10.48550/arXiv:1303.3997v2

[B31] Li H. (2021). Wgsim. Github repository. Available online at: https://github.com/lh3/wgsim.

[B32] Li H. Durbin R. (2009). Fast and accurate short read alignment with Burrows-Wheeler transform. Bioinformatics 25, 1754–1760. doi: 10.1093/bioinformatics/btp324 19451168 PMC2705234

[B33] Li H. Handsaker B. Wysoker A. Fennell T. Ruan J. Homer N.. (2009). The sequence alignment/map format and SAMtools. Bioinformatics 25, 2078–2079. doi: 10.1093/bioinformatics/btp352 19505943 PMC2723002

[B34] Li Y. Cao X. L. Zhu Y. Yang X. M. Zhang K. N. Xiao Z. Y.. (2019). Osa-miR398b boosts H_2_O_2_ production and rice blast disease-resistance via multiple superoxide dismutases. New Phytol. 222, 1507–1522. doi: 10.1111/nph.15678 30632163 PMC6593823

[B35] Li Q. Fu J. Qin X. J. Yang W. Qi J. J. Li Z. G.. (2020). Systematic analysis and functional validation of citrus pectin acetylesterases (CsPAEs) reveals that CsPAE2 negatively regulates citrus bacterial canker development. Int. J. Mol. Sci. 21, 14. doi: 10.3390/ijms21249429 PMC7764809 33322321

[B36] Lin N. C. Abramovitch R. B. Kim Y. J. Martin G. B. (2006). Diverse AvrPtoB homologs from several *Pseudomonas syringae* pathovars elicit Pto-dependent resistance and have similar virulence activities. Appl. Environ. Microbiol. 72, 702–712. doi: 10.1128/AEM.72.1.702-712.2006 16391110 PMC1352197

[B37] Luo Q. Liu W. W. Pan K. D. Peng Y. L. Fan J. (2017). Genetic interaction between Arabidopsis *Qpm3.1* locus and bacterial effector gene *hopW1-1* underlies natural variation in quantitative disease resistance to *Pseudomonas* infection. Front. Plant Sci. 8. doi: 10.3389/fpls.2017.00695 PMC5415610 28523008

[B38] Martin M. (2011). Cutadapt removes adapter sequences from high-throughput sequencing reads. EMBnet.journal 17, 10–12. doi: 10.14806/ej.17.1

[B39] Martin G. B. Brommonschenkel S. H. Chunwongse J. Frary A. Ganal M. W. Spivey R.. (1993). Map-based cloning of a protein kinase gene conferring disease resistance in tomato. Science 262, 1432–1436. doi: 10.1126/science.7902614 7902614

[B40] Mazo-Molina C. Mainiero S. Hind S. R. Kraus C. M. Vachev M. Maviane-Macia F.. (2019). The *Ptr1* locus of *Solanum lycopersicoides* confers resistance to race 1 strains of *Pseudomonas syringae* pv. *tomato* and to *Ralstonia pseudosolanacearum* by recognizing the type III effectors AvrRpt2 and RipBN. Mol. Plant-Microbe Interact. 32, 949–960. doi: 10.1094/MPMI-01-19-0018-R 30785360

[B41] Mazo-Molina C. Mainiero S. Haefner B. J. Bednarek R. Zhang J. Feder A.. (2020). Ptr1 evolved convergently with RPS2 and Mr5 to mediate recognition of AvrRpt2 in diverse Solanaceous species. Plant J. 103, 1433–1445. doi: 10.1111/tpj.14810 32391580

[B42] Menda N. Strickler S. R. Edwards J. D. Bombarely A. Dunham D. M. Martin G. B.. (2014). Analysis of wild-species introgressions in tomato inbreds uncovers ancestral origins. BMC Plant Biol. 14, 16. doi: 10.1186/s12870-014-0287-2 25348801 PMC4219026

[B43] Park H. J. Kim W. Y. Park H. C. Lee S. Y. Bohnert H. J. Yun D. J. (2011). SUMO and SUMOylation in plants. Molecules Cells 32, 305–316. doi: 10.1007/s10059-011-0122-7 21912873 PMC3887640

[B44] Pearson W. R. (2013). An introduction to sequence similarity (homology) searching. Curr. Protoc. Bioinf. 3, 3.1.1–3.1.8. doi: 10.1002/0471250953.bi0301s42 PMC3820096 23749753

[B45] Pedley K. F. Martin G. B. (2003). Molecular basis of Pto-mediated resistance to bacterial speck disease in tomato. Annu. Rev. Phytopathol. 41, 215–243. doi: 10.1146/annurev.phyto.41.121602.143032 14527329

[B46] Pilowsky M. Zutra D. (1982). Screening wild tomatoes for resistance to bacterial speck pathogen (*Pseudomonas* tomato). Plant Dis. 66, 46–47. doi: 10.1094/PD-66-46

[B47] Pitblado R. E. MacNeill B. H. (1983). Genetic basis of resistance to *Pseudomonas syringae* pv. tomato Field tomatoes. Can. J. Plant Pathol. 5, 251–255. doi: 10.1080/07060668309501606

[B48] Poland J. A. Balint-Kurti P. J. Wisser R. J. Pratt R. C. Nelson R. J. (2009). Shades of gray: the world of quantitative disease resistance. Trends Plant Sci. 14, 21–29. doi: 10.1016/j.tplants.2008.10.006 19062327

[B49] Ramu V. S. Dawane A. Lee S. Oh S. Lee H. K. Sun L.. (2020). Ribosomal protein QM/RPL10 positively regulates defense and protein translation mechanisms during nonhost disease resistance. Mol. Plant Pathol. 21, 1481–1494. doi: 10.1111/mpp.12991 32964634 PMC7548997

[B50] Ronald P. C. Salmeron J. M. Carland F. M. Staskawicz B. J.. (1992). The cloned avirulence gene *AvrPto* induces disease resistance in tomato cultivars containing the *Pto* resistance gene. J. Bacteriology 174, 1604–1611. doi: 10.1128/jb.174.5.1604-1611.1992 PMC206556 1537802

[B51] Salmeron J. M. Oldroyd G. E.D. Rommens C. M.T. Scofield S. R. Kim H. S. Lavelle D. T.. (1996). Tomato *Prf* is a member of the leucine-rich repeat class of plant disease resistance genes and lies embedded within the *Pto* kinase gene cluster. Cell 86, 123–133. doi: 10.1016/S0092-8674(00)80083-5 8689679

[B52] Sato S. Tabata S. Hirakawa H. Asamizu E. Shirasawa K. Isobe S.. (2012). The tomato genome sequence provides insights into fleshy fruit evolution. Nature 485, 635–641. doi: 10.1038/nature11119 22660326 PMC3378239

[B53] Schreiber K. J. Baudin M. Hassan J. A. Lewis J. D. (2016). Die another day: molecular mechanisms of effector-triggered immunity elicited by type III secreted effector proteins. Semin. Cell Dev. Biol. 56, 124–133. doi: 10.1016/j.semcdb.2016.05.001 27166224

[B54] Schreiber K. J. Chau-Ly I. J. Lewis J. D. (2021a). What the wild things do: mechanisms of plant host manipulation by bacterial type III secreted effector proteins. Microorganisms 9, 48. doi: 10.3390/microorganisms9051029 PMC8150971 34064647

[B55] Schreiber K. J. Hassan J. A. Lewis J. D. (2021b). Arabidopsis Abscisic Acid Repressor 1 is a susceptibility hub that interacts with multiple *Pseudomonas syringae* effectors. Plant J. 105, 1274–1292. doi: 10.1111/tpj.15110 33289145

[B56] Schreiber K. J. Lewis J. D. (2021). Identification of a putative DNA-binding protein in Arabidopsis that acts as a susceptibility hub and interacts with multiple *Pseudomonas syringae* effectors. Mol. Plant-Microbe Interact. 34, 410–425. doi: 10.1094/MPMI-10-20-0291-R 33373263

[B57] Singh V. Sinha P. Obala J. Khan A. W. Chitikineni A. Saxena R. K.. (2022). QTL-seq for the identification of candidate genes for days to flowering and leaf shape in pigeonpea. Heredity 128, 411–419. doi: 10.1038/s41437-021-00486-x 35022582 PMC9177671

[B58] Sobol G. Chakraborty J. Martin G. B. Sessa G. (2022). The emerging role of PP2C phosphatases in tomato immunity. Mol. Plant-Microbe Interact. 35, 737–747. doi: 10.1094/MPMI-02-22-0037-CR 35696659

[B59] Son S. Park S. R. (2023). Plant translational reprogramming for stress resilience. Front. Plant Sci. 14, 12. doi: 10.3389/fpls.2023.1151587 PMC9998923 36909402

[B60] Su X. Wang B. A. Geng X. L. Du Y. F. Yang Q. Q. Liang B.. (2021). A high-continuity and annotated tomato reference genome. BMC. Genomics 22, 12. doi: 10.1186/s12864-021-08212-x 34911432 PMC8672587

[B61] Sun W. Y. Zhao W. Y. Wang Y. Y. Pei C. C. Yang W. C. (2011). Natural variation of *pto* and *fen* genes and marker-assisted selection for resistance to bacterial speck in tomato. Agric. Sci. China 10, 827–837. doi: 10.1016/S1671-2927(11)60068-0

[B62] Takagi H. Abe A. Yoshida K. Kosugi S. Natsume S. Mitsuoka C.. (2013). QTL-seq: rapid mapping of quantitative trait loci in rice by whole genome resequencing of DNA from two bulked populations. Plant J. 74, 174–183. doi: 10.1111/tpj.12105 23289725

[B63] Takei H. Shirasawa K. Kuwabara K. Toyoda A. Matsuzawa Y. Iioka S.. (2021). *De novo* genome assembly of two tomato ancestors, *Solanum pimpinellifolium* and *Solanum lycopersicum* var. *cerasiforme*, by long-read sequencing. DNA Res. 28, 9. doi: 10.1093/dnares/dsaa029 PMC7934570 33475141

[B64] Tang X. Y. Frederick R. D. Zhou J. M. Halterman D. A. Jia Y. L. Martin G. B. (1996). Initiation of plant disease resistance by physical interaction of AvrPto and Pto kinase. Science 274, 2060–2063. doi: 10.1126/science.274.5295.2060 8953033

[B65] Thapa S. P. Miyao E. M. Davis R. M. Coaker G. (2015). Identification of QTLs controlling resistance to *Pseudomonas syringae* pv. *tomato* race 1 strains from the wild tomato, *Solanum habrochaites* LA1777. Theor. Appl. Genet. 128, 681–692. doi: 10.1007/s00122-015-2463-7 25634105

[B66] Valenzuela M. Fuentes B. Alfaro J. F. Gálvez E. Salinas A. Besoain X.. (2022). First Report of Bacterial Speck Caused by *Pseudomonas syringae* pv. *tomato* Race 1 Affecting Tomato in Different Regions of Chile. Plant Dis. 106, 1979–1979. doi: 10.1094/PDIS-11-21-2436-PDN

[B67] van den Burg H. A. Kini R. K. Schuurink R. C. Takken F. L.W. (2010). Arabidopsis small ubiquitin-like modifier paralogs have distinct functions in development and defense. Plant Cell 22, 1998–2016. doi: 10.1105/tpc.109.070961 20525853 PMC2910984

[B68] Wang X. Gao L. Jiao C. Stravoravdis S. Hosmani P. S. Saha S.. (2020). Genome of *Solanum pimpinellifolium* provides insights into structural variants during tomato breeding. Nat. Commun. 11, 11. doi: 10.1038/s41467-020-19682-0 33199703 PMC7670462

[B69] Ward N. Moreno-Hagelsieb G. (2014). Quickly finding orthologs as reciprocal best hits with BLAT, LAST, and UBLAST: how much do we miss? PloS One 9, 6. doi: 10.1371/journal.pone.0101850 PMC4094424 25013894

[B70] Widjaja I. Lassowskat I. Bethke G. Eschen-Lippold L. Long H. H. Naumann K.. (2010). A protein phosphatase 2C, responsive to the bacterial effector AvrRpm1 but not to the AvrB effector, regulates defense responses in Arabidopsis. Plant J. 61, 249–258. doi: 10.1111/j.1365-313X.2009.04047.x 19843314

[B71] Xin X. F. Kvitko B. He S. Y. (2018). *Pseudomonas syringae*: what it takes to be a pathogen. Nat. Rev. Microbiol. 16, 316–328. doi: 10.1038/nrmicro.2018.17 29479077 PMC5972017

[B72] Yunis H. Bashan Y. Henis Y. (1980). Two sources of resistance to bacterial speck of tomato caused by *Pseudomonas tomato* . Plant Dis. 64, 851–852. doi: 10.1094/PD-64-851

[B73] Zamir D. (2001). Improving plant breeding with exotic genetic libraries. Nat. Rev. Genet. 2, 983–989. doi: 10.1038/35103590 11733751

