# Supplementary material for: Solanum pimpinellifolium exhibits complex genetic resistance to Pseudomonas syringae pv. tomato
Source: Front Plant Sci. 2024 Oct 23;15:1416078. doi: 10.3389/fpls.2024.1416078 (PMC11537850; doi:10.3389/fpls.2024.1416078)
Supplement: Supplementary file 1 [file DataSheet1.docx]

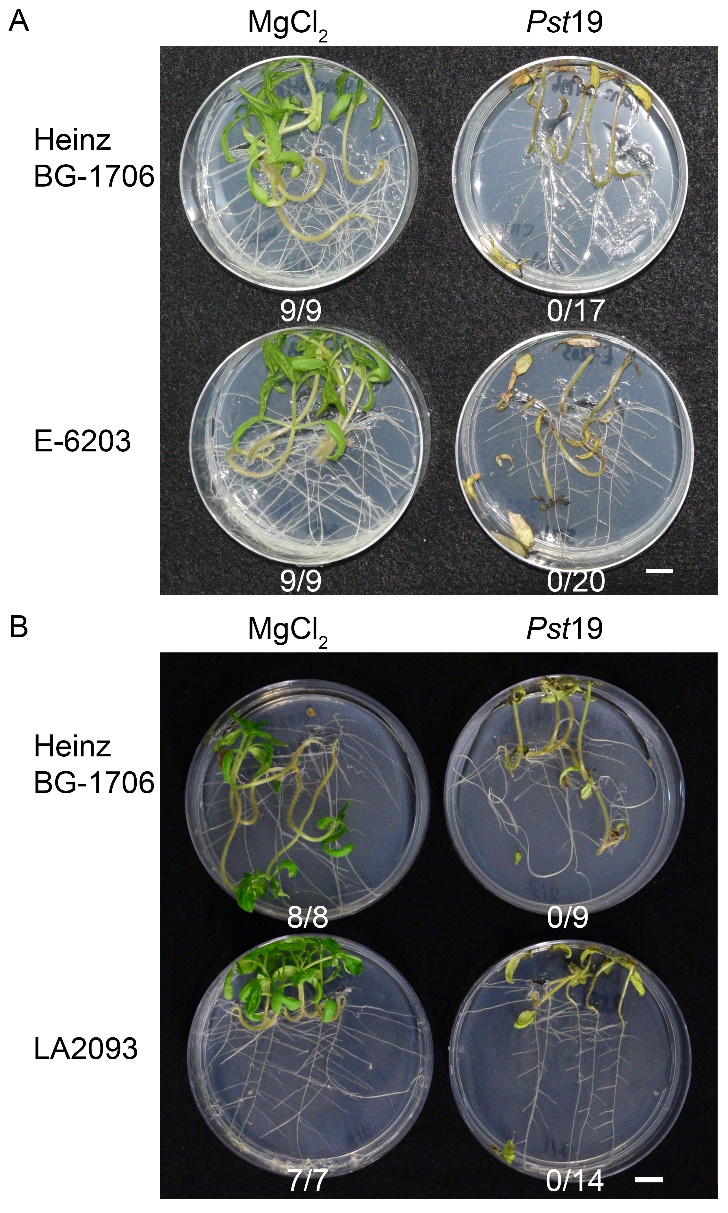


**Supplemental** **Figure S1.** Tomato cultivars Heinz BG-1706 and E-6203 (A) and *S. pimpinellifolium* LA2093 (B) are susceptible to *Pst*19. All seedlings show disease symptoms 10-14 days after being flooded with *Pst*19. The number of surviving plants is indicated over the total number of tested plants.

**Supplemental Tables**

| **Table S1.** Frequency of *Pst*19 resistance in seedlings from recombinant inbred backcross lines | | | | |
| --- | --- | --- | --- | --- |
| **Inbred Backcross Line** | **# Susceptible Seedlings** | **# Resistant Seedlings** | **Total Seedlings Tested** | **Overall Resistance (%)** |
| LA4139 | 20 | 0 | 20 | 0% |
| LA4140 | 21 | 0 | 21 | 0% |
| LA4141 | 26 | 0 | 26 | 0% |
| LA4142 | 10 | 0 | 10 | 0% |
| LA4143 | 51 | 2 | 53 | 3.77% |
| LA4144 | 20 | 3 | 23 | 13.04% |
| LA4145 | 32 | 0 | 32 | 0% |
| LA4146 | 20 | 0 | 20 | 0% |
| LA4147 | 27 | 3 | 30 | 10.00% |
| LA4148 | 24 | 3 | 27 | 11.11% |
| LA4149 | 32 | 3 | 35 | 8.57% |
| LA4150 | 19 | 4 | 23 | 17.39% |
| LA4151 | 15 | 0 | 15 | 0% |
| LA4152 | 15 | 0 | 15 | 0% |
| LA4153 | 20 | 0 | 20 | 0% |
| LA4154 | 18 | 0 | 18 | 0% |
| LA4155 | 15 | 0 | 15 | 0% |
| LA4156 | 22 | 14 | 36 | 38.88% |
| LA4157 | 17 | 0 | 17 | 0% |
| LA4158 | 25 | 0 | 25 | 0% |
| LA4159 | 10 | 0 | 10 | 0% |
| LA4160 | 19 | 0 | 19 | 0% |
| LA4161 | 16 | 0 | 16 | 0% |
| LA4162 | 17 | 0 | 17 | 0% |
| LA4163 | 16 | 0 | 16 | 0% |
| LA4165 | 14 | 0 | 14 | 0% |
| LA4166 | 16 | 0 | 16 | 0% |
| LA4167 | 19 | 2 | 21 | 9.52% |
| LA4168 | 21 | 16 | 37 | 43.24% |
| LA4169 | 15 | 0 | 15 | 0% |
| LA4170 | 25 | 0 | 25 | 0% |
| LA4171 | 12 | 0 | 12 | 0% |
| LA4172 | 24 | 0 | 24 | 0% |
| LA4173 | 24 | 3 | 27 | 11.11% |
| LA4174 | 23 | 0 | 23 | 0% |
| LA4176 | 13 | 0 | 13 | 0% |
| LA4177 | 25 | 0 | 25 | 0% |
| LA4178 | 25 | 0 | 25 | 0% |
| LA4179 | 16 | 0 | 16 | 0% |
| LA4180 | 13 | 0 | 13 | 0% |
| LA4181 | 11 | 0 | 11 | 0% |
| LA4182 | 15 | 0 | 15 | 0% |
| LA4183 | 9 | 0 | 9 | 0% |
| LA4184 | 10 | 0 | 10 | 0% |
| LA4185 | 14 | 0 | 14 | 0% |
| LA4186 | 8 | 0 | 8 | 0% |
| LA4187 | 19 | 0 | 19 | 0% |
| LA4188 | 10 | 0 | 10 | 0% |
| LA4189 | 17 | 1 | 18 | 5.56% |
| LA4190 | 9 | 0 | 9 | 0% |
| LA4191 | 9 | 0 | 9 | 0% |
| LA4192 | 20 | 1 | 21 | 4.76% |
| LA4193 | 9 | 0 | 9 | 0% |
| LA4194 | 21 | 0 | 21 | 0% |
| LA4195 | 25 | 0 | 25 | 0% |
| LA4196 | 21 | 1 | 22 | 4.55% |
| LA4197 | 25 | 0 | 25 | 0% |
| LA4198 | 10 | 1 | 11 | 9.09% |
| LA4199 | 30 | 2 | 32 | 6.25% |
| LA4200 | 30 | 1 | 31 | 3.23% |
| LA4201 | 8 | 0 | 8 | 0% |
| LA4202 | 15 | 1 | 16 | 6.25% |
| LA4203 | 24 | 2 | 26 | 7.69% |
| LA4204 | 17 | 2 | 19 | 10.53% |
| LA4205 | 14 | 2 | 16 | 12.5% |
| LA4206 | 10 | 0 | 10 | 0% |
| LA4207 | 17 | 2 | 19 | 10.53% |
| LA4208 | 11 | 8 | 19 | 42.11% |
| LA4210 | 10 | 0 | 10 | 0% |
| LA4211 | 7 | 0 | 7 | 0% |
| LA4212 | 10 | 0 | 10 | 0% |
| LA4213 | 21 | 0 | 21 | 0% |
| LA4214 | 28 | 1 | 29 | 3.45% |
| LA4215 | 6 | 0 | 6 | 0% |
| LA4216 | 24 | 14 | 38 | 36.84% |
| LA4217 | 9 | 0 | 9 | 0% |
| LA4218 | 22 | 0 | 22 | 0% |
| LA4219 | 9 | 0 | 9 | 0% |
| LA4220 | 8 | 0 | 8 | 0% |
| LA4221 | 21 | 0 | 21 | 0% |
| LA4223 | 19 | 0 | 19 | 0% |
| LA4224 | 9 | 0 | 9 | 0% |
| LA4225 | 2 | 0 | 2 | 0% |
| LA4226 | 24 | 0 | 24 | 0% |
| LA4227 | 35 | 0 | 35 | 0% |
| LA4228 | 37 | 5 | 42 | 11.90% |
| LA4229 | 20 | 1 | 21 | 4.76% |
| Inbred backcrossed lines were derived from a cross between E-6203 x *S. pimpinellifolium* LA1589 (Doganlar et al. 2002). | | | | |

| **Table S2.** LA1589^1^ and Heinz BG-1706 locations and Wilcoxon Mann-Whitney test for significance of IBL markers used to define chromosomal intervals in LA1589 | | | | | |
| --- | --- | --- | --- | --- | --- |
| **Chr #** | **IBL marker** | **SGN marker** | **Significance of marker linked to resistance^2^** | **Heinz marker coordinates^3^** | **LA1589 marker coordinates^4^** |
| 5 | CT101 | SGN-M384 | ** | SL4.0ch05:897017…897148 | Spim0.1ch05: 121112...124083 |
| 5 | TG441 | SGN-M102 | * | SL4.0ch05:1997864…1997884 | Spim0.1ch05: 1159752...1160210 |
| 5 | CT167 | SGN-M188 | ns | SL4.0ch05:2721438…2721899 | Spim0.1ch05: 1915173…1915640 |
| 6 | CT216 | SGN-M516 | ** | SL4.0ch06:730940…730959 | Spim0.1ch06: 702949…703437 |
| 6 | TG178 | SGN-M143 | ns | SL4.0ch06:22112109…22112568 | Spim0.1ch06: 22761978…22762437 |
| 8 | TG302 | SGN-M111 | ns | SL4.0ch08:56766398…56766417 | Spim0.1ch08: 57274175...57275344 |
| 8 | TG201 | SGN-M391 | *** | SL4.0ch08:61445780…61446278 | Spim0.1ch08: 62316597...62392315 |
| 8 | CT265 | SGN-M673 | ** | SL4.0ch08:61783768…61784009 | Spim0.1ch08: 62698577…62698818 |
| 8 | CT68 | SGN-M44 | ns | SL4.0ch08:63905392…63905956 | Spim0.1ch08: 64810648...64811212 |
| ^1^LA1589 PacBio genome sequence (Alonge et al. 2020)  ^2^Sigificance of markers determined using Wilcoxon Mann-Whitney test with the following p-values. *** p<0.01 highly significant, ** p<0.05 significant, * p<0.1 suggestive, p>0.1 not significant (ns).  ^3^From SL4.0_SGNmarker_sequences in ITAG4.0 Release obtained from https://solgenomics.net/ftp/tomato_genome/annotation/ITAG4.0_release/  ^4^If marker mapped to more than one region, then region with smallest e-value was selected | | | | | |

| **Table S3.** Sequences of RFLP markers | | | |
| --- | --- | --- | --- |
| Chr^1^ | IBL Marker | Forward Sequence^2^ | Reverse Sequence^2^ |
| 5 | CT101 | CGGAATTCCTTTTTTTTTTTTTTTTTTTGAATATGAAATGCTGAAAAAAAATTCAACCTTGCGTGTTCTTGAACTAAACAATAATCTGATTGACTATTCTGGATTTTCAGGTCTTGCTGGATCACTTCTTGAGAATAAAACTTTACAATCCTTGCATCTCAACGGCAATTATGGTGGTGCTTTAGGGGCTGCAGCACTGGCTAAAGGCTTGGAGAGCAATAAGTCCTTGAGGGAACTTTATTTGCAGGGGAACTCTGTTGGCGATGAAGGAGTACGGGCATTAATCTCTGGCCTGTCTTTGCGTAAAGGGAAACTTGTCTTACTTGACCTTGCCAACAATTCAATTACTGCAAGAGGAGCATTTCATGTTGCCGAATACGTCAAAAAAAGCAAAAGCTTACTGTGGTTGAATCTCTATATGAATGACATTAAAGATGAGGGAGCTGAAAAGATTGCAGAAGCTTTGAAGGAGAACCGCTCGATAACTAATGTTGATCTTG | TTTTTTTTTTTTTTTGAATATGAAATGGTGAATAGAAGTAATCTCTATATACTTGCCACTAATGTTTCAGAAACGGAATGTTATTTATCAAAATTTCCACCGTCCCCCCGCTCAAAATGATTCAGTCACAAAGGGGAGCTGAGGTCGGACTCTTAGGAATATCATTGGTAAAAGAAAAAAAAATACTCACCTATGCAGCCCTGCCCCAACCTCAAGAATGAAAGCAGGGAGAAAAATAAATCTGATCTAAGGAATGTGGTATTTTAAAACATAACAGCAAGCTCCTTCTCAGTCATCTCAAATACATGGTCTCTTGCATCTGTGAGAGCTGTCTGTCCTAATTTGGTGAGGAAGTTACTGGCAAGATTTAAAGATGTGAGTCTAACATCTTCATTTGCTTTAAGTGCTTGAGCGATAGAAAAAGCTCCTTCATCTCTAATTTCATTGAACCCTAAATTGAGTGTCATCAAAGATTCATTGACTACTT |
| 5 | TG441 | TGTATTGAATAATGTCAGCATAGGCTTTTCCAAGCCTGCTCTGAGGTAAACTCTCAGTTCTCTGATTGTCTCCTTGATCCTGAGGCAAAAAGATGTCATACAACATTTTGTATCAGTTGGAGGGGGAAGAGGTCAGAGGAGAGGAAGAATGAACACACACAACTGAAGGCACAACAAAATGCCAACATACAATAGATGAAGTTGATGTGGTGACTCGTTGACCAAGTGGACTATCCGAAAACATGGTTTCACTTCTTGCATCATATTTTCCTCTGCGAGAGCGTCCCCTCGTAATTATTGCTTTCAGCCCAGTAATCCAAACCTCAGCTTCATCCTTGTCTTTACAGATCTACACAGAATTATCATAACAGTTACTAGATTATTTTTTTAAGACTTGTAAATATCAAAATGTCATTTTTCCCGACCGGAAAGAGTACTTATTTTCATTGAGAAGACAAA | TGGTATGGTACCACGACAAAGAAGAAAAACAGCTTGAACTGTGTCATGTATCAAGGATTATTCCTGGACAGAGAACTGTGAGTTTGAGTATTCCCACATCTTTCTTCCCTTATAAAGGAAATTTTTTCCCTCAAGTTCCATTTGAATGGAAGATTTGCAGGTTTTACTCTTTAAGAGAAGGTGGATTAAATTTTCTGATCATTGATCAGCAAGAGTAATTTCTGCATCGAGTTCTTTGTTGTGCTCTATGACTGATTTGTAAAGACCATATATTATAGTTCACAAGGGCATTGCTATTCTTATGTCTGTCGAGTTTAGATCCTTTTTCTGTGTGGTTTATACTCCTCTTCCTCTTGTGCTAGACCTTTATTTTCAAGTGGCTGACCAAAACAGTGGTAATTTTCTCCTCTTCAGGCAATATTCCAGCGGTATCCTCGACCTGAAAAGGAGTATCAATCATTTTCTC |
| 5 | CT167^3^ | ATTAGCATTTGTGCTGGATTGTACTGTTGTTGTTGGCCTCCGTTGTTCATGTTCATTAGCATCGAGGCAGAGGAAGGATGATGATGACCACCGGTGGATAAAGATCCCGGTATAGCATTGTTTTGATGACCTTGAAATCCACCCGCGTTCAAGAATGCAGCAACGTTGTTGTTATTGTTAGTAGTAGTAGTAGTAGTTCCATTGTTGTTATTCCCATGAAAACCAGCAAGATTCATCATTGAGTTTAAGTCGTTTCCTCCCACCATTCCAATTTTCGCAGCCTCGTTGAGGTTGACATTTGGACCTAATTGAGCAGCAGAAGCAGCACCATTCATGTTCATTTTCATAGCATGAATTGCTCTTTGGTCAATGCCACCGCCTCCGGGATTTGCCTTCATTGGCATATTCGGACTAGGGCCAGTTTTCTTGCCATGAGCAGCAACATTGTTGTTGTTACCTTTC | Not Available |
| 6 | CT216 | CTTTTTTTTTTTTTCAAACAACACCAAAAAAAAGTTGAGTATTCACAGCTTAAGTTTTCTTTCAATTGCTATAAAAAAAAACAATGGTCTTTCTATTACTTATAATTACTGCTACAAATTCAACAAAATGACACTAAAAATGTATTGAATTTCATATCAAAGCCTTCTCTATTTACATATTCCCAACAAAATGGCATCTTTTCTCTATGCCATATAGGCCGCTCTCTTATCCATCAATCTTCTACTTCTAGTCGACTGCTCTAGAACCAAACTTTCTCTTTTTTTAGTGCTCCCTTCCCACTCAACAGGTAATAATTTAAAGAACGACATTATTGTCTTCTTCTTCTAATCGACTGCTCTACCAATCACACATTGCCTTACACTTGGCATCGGGGGATATCGTCTTCTGCTTCTAGTCGACTGCTCTATAAAAATAATAATTGAATATTGGCGGTTTCCGACTAAGGAATTACCATCTTGTTACTCCATTACGTTCT | TTTTTTTTTCTCTTAGGAAACCTTTTTTATATTCTCCGGCGAGCCAAATCGGAGCATAATTTGGGGGTTTTGAGTTATTGGATCTAACCGGGTTTATTGGGTTGAGTTTTGACTGATCTCCGGCGACTGCAGATGTCTTCGTACTGGTGTTATCGGTGTACAAGGTTTGTTAGAATTTCAGTTGGAAACGATGTTGTTTGTCCTCACTGTGATAGTGGATTTATAGAAGTTGCTGAGGGAAATATTGGGTCGCCGGAATCTCGCCGGAGGTTTCCCATGTGGAACGAACGGCCAGAGTCAGATCGGAGTGTGAACATGGGTTCTCGACGGAGCAGGCGTAATCGGGGTGATAGATCTCCGTTTAATCCGGTTATTGTTCTTCGTAGTCCATCTGAAGCACCTGGGGAGGATGAAGCTGCTGCAGCTGCAGAGGAGCGGAGTTATGAGCTTTATTATGATGATGGTGAAGGTTCAGGTCTCAGGCCGTTGCCGCCGACGATGTCGGAGTTTTTGATGGGAT |
| 6 | TG178^3^ | AATACACCGTTTTCGGCAGGAGAGCAATTCCCCCTACAAACAAAGCCATCAATCATCATGCCTGCACTTGTTGTAACTGAAGAAACAATGGCCTCCGAGCTATCCAAGAAAAAAATGAAGAAGACATCCCAAACAGATATTGAAACTCCTACTGATAAGAAAACCAAAGAGAAAAAGTCAAAAAAATCCAAGATTGAGTCTGGGTCTGATTCAGAGGACGCTAAAAGGAGTAAAAAGAAAGAGAAAAAGAGAAAAGCTTTGGACTTGGATGGTGAGAAGAGTGATACGAGCTCAGAGATATGTGAGCCCGTAGATTTGAAGAAGAATAAAAAGGCTAAATTCGACGACGAACAAGTGATGGTTGAGAAGAAAGTCGAGGATCCTAATGCTTTGTCTAATTTTAGGATTTCAAAGCCTTTAAAAGAGGCTTTGAATTCTAAGGGAATAGAAGCACTTTTTCCTATTCA | Not Available |
| 8 | TG302 | ATGGTTGCATTACTCAGTCATTGCACTCTCCGGGTGGCTATTACATTTTTGAAAGGAGTAATTATTTCCCGCAAGAGAAGCACTTCCCTTATGTATAGAAATCAGTGACGTAAAATTTATAATCAACATAAAACTCTAAAGTTTGGTTCTGGTGAAGTAACTGGAGTTAAATAATCCTACTAACACTCATATCAGAGGGTTGGCTCCAAACAAATTTCTCGGTCATGAAAAACACCCATATAAGAGAAAGAATAGACATCCTTATTTTTGGGGAAGGATTTTGGTTCCTATCAGTGAAATTTGGAATCATAGTTATAAGATGAAAAAAATGAGTGGAAAATTACTTATTAAAAAAGAAAACAGGGGTGATACCAGCTTAGATGGATCGATTTAAGTGCTATAAGATGTAGAAGTATTTGCAAGGTGAAAGGACTCTCCCGCGAATTTATTAAAATAAATTTACATTTATTGGTCACAA | AAAGAAGCAAGAACCATTTTAATTAGGAAAGCATCTAGCTGAAATTAGCGGCCAGAGGCACCACCTCTTGCAAGAAAAGATACTATAACCCTAGCTAGAAAAAGTGCAAATTTTTATGTAAACAGCATTGTCCAACAAAAATAAAGGAAGACTAAAAACAACATAAATCTAACTTGTTTGTCAGAACTAGATATCTCAAATAAATGAAAGTTTGTCTTATTTCTACTAATTATCATCTGGCAACTGGCATTTCTATCCTAGTTGAATAACCTGCAAGTTTATGAGCAAATCTGATTGTTATCTTTTTATTTCTTTTTGGTGTAAATTGAATGAGTAAATGATTAGGAACTCCAATTACAGTTCTTAGAAGCTTTTGCATATGCTAGTCTTGGGACTCCTCCTTTTCTTTCCTTTTTTCTGTTCTCTTTTGGATGTAATGACAGCACTTTCTTAGTGCTAATTTTGACATCAATAATATTAACCCTTCATAAA |
| 8 | TG201 | AATACCAAGATTTAGAAAGCTGGTATCTTTCTTTCTTGCCTACAACAACCTCCGTTAGTTCTCGTGAGGCACCACGTTTGATATATTCCTATCGAAATGTCTTAACAGGCTTTGCAGCTAAGTTATCAGAAGAAGATATAAAGGAAATGGAAAAAAAGGAAGGATTTGTTTCTGCACGCCCCCAGCAGTTTGTTAGTTTACACACCACACATAGTGTCAATTTCTTGGGATTGCAACAGAACATGGGGTTCTGGAAAGACTCTAATTATGGGAAAGGTGTGATCATCGGAGTTCTAGACACTGGAATTCTTCCTGACCATCCTTCATTTAGTGACGTTGGGATGCCTACCCCACCTGCTAAGTGGAAAGGAGTTTGTGAGTCAAATTTTATGAACAAGTGTAACAAAAAGCTCATTGGAGCGAGGTCTTACCAACTTGGCAATGGTTCCCCGATAGATGGTAATGGACACGGTACACACACAGCAAGCACAGCTGCAGGCATG | CTCACTATAGGGAGACAGCCTGCATGCCTACAGCTGTGCTTGCTGTGTGTGTACCGTGTCCATTACCATCTATCGGGGAACCATTGCCAAGTTGGTAAGACCTCGCTCCAATGAGCTTTTTGTTACACTTGTTCATAAAATTTGACTCACAAACTCCTTTCCACTTAGCAGGTGGGGTAGGCATCCCAACGTCACTAAATGAAGGATGGTCAGGAAGAATTCCAGTGTCTAGAACTCCGATGATCACACCTTTCCCATAATTAGAGTCTTTCCAGAACCCCATGTTCTGTTGCAATCCCAAGAAATTGACACTATGTGTGGTGTGTAAACTAACAAACTGCTGGGGGCGTGCAGAAACAAATCCTTCCTTTTTTTCCATTTCCTTTATATCTTCTTCTGATAACTTAGCTGCAAAGCCTGTTAAGACATTTCGATAGGAATATATCAAACGTGGTGCCTCACGAGAACTAACGGAGGTTGTTGTAGGCAAGAAAGAAAGATACCAGCTTTCTAAATCTTGGTATT |
| 8 | CT265^3^ | Not available | TTTTTTTTTGCTAGGAAGGGTCAGAGATTTTCTCTTCCCGATGAGAGGCGGCCTCCTGTGGAGTTCATTGCCTTCTCCAATTGCTTGCTTGGTTGGACCGGGGGTGCCGATGCTTTGACAAGGGGGATTTTTACAGGCCACCTTTTGCCCCTATAATGCCTCGAGCTACTTTCCTGGAGTATGGGAATGATCAAGCTTTTTTAGAATTAATTCCCAGTGGTTGAATAGCTGCTGTATTTGTCGAACCTCTCC |
| 8 | CT68 | CAGATCTACAGATGGAAGATCAATTGCAGACGATTGTCCGAGATCACTTGTAGAGCTGGAATCAATGTTACAAGCCAGAAAAGAAGCCTCCTTCAAGCGGGAAAAATCCCTTGCTCATGCTTTTACTCAACAGGAATTGGATGAGATGGATGCTGTTTGTAGTGAAGAAAGAAATGAAAGGGAATTAGAAGAGACAGCGAATTGGCTAGACGAGTGGATGTCATCAAAGCAATGGAACAGAGGTTCATTTGACAGAACAGACTCTATAAAGACTGTTGAGAAGGACACGGCTAAGCCATATTGTAACATGGTTCTCAATGCTCTCAAGACACCACACTCTAGCCCACTTCGCAGACGGGCTAATAGTCCTCATTATACTGCTAATTCTCCCCATCACCAGAGATCATCACATTACGATTACTCGGCAATTCAACCACCAGCCACCCCACCCCCTTGTCAACCAGAACCTCTTCAAATGC | CAGATCTAGAGATGGAAGCTCAATTGCAGACGATTGTCCGAGATCACTTGTAGAGCTGGAATCAATGTTACAAGCCAGAAAAGAAGCCTCCTTCAAGCGGGAAAAATCCCTTGCTCATGCTTTTACTCAACAGGAATTGGATGAGATGGATATTGTTTGTAGTGAAGAAAGAAATGAAAGGGAATTAGAAGAGACAGCGAATTGGCTAGACGAGTGGATGTCATCAAAGCAATGGAACAGAGGTTCATTTGACAGAAGAGACTCTATAAAGACTGTTGAGATGGACACGGCTAAGCCATATTGTAACATGGTTCCAAATGCTCGAAGATCACAACACTCTAGCCCACTTCACAGACAGGCTAGTAGTCCTCATTATACTGCTAATTCTCCCCATCACCAGAGATCATCACATTACAATTACTCGGCAATTCAACCACCAGCCACCCCACCCCCTTGTCAACCAAAACCTCTTCAAATGCGCTCAACAAGCCCACGTAAAAGCCAATCAACTGCAAACAC |
| Forward and reverse sequences were blasted to LA1589 PacBio genome sequence (Alonge et al. 2020) to delineate regions associated with LA1589 resistance. CD40, CT111, TG330, TG505, TG201 and CT265 (all on chr 8) sequences were not available. The next closest marker was used to delimit regions.  ^1^Chr indicates chromosome.  ^2^<https://solgenomics.net>. The RFLPs were sequenced from either end and the sequences are non-overlapping.  ^3^Only one sequence was available. | | | |

| **Table S4.** Presence of individual homozygous or heterozygous LA1589 markers linked to *Pst*19 resistance in eight Inbred Backcross Lines | | | | | | |
| --- | --- | --- | --- | --- | --- | --- |
| Line# | Markers^1^ and chromosome numbers (Chr) | | | | | |
|  | CT101**  Chr 5 | CT441*  Chr 5 | CT216**  Chr 6 | TG176*  Chr 8 | TG201***  Chr 8 | CT265**  Chr 8 |
| LA4144 | - | - | HM | HM | HM | HM |
| LA4156 | - | - | HM | - | - | - |
| LA4168 | - | - | - | HM | - | HM |
| LA4173 | - | - | HM | HM | HM | HM |
| LA4208 | HM | HM | HM | - | HM | HET |
| LA4216 | - | - | HET | HM | - | - |
| LA4148 | - | - | - | - | HM | HM |
| LA4152 | - | - | - | - | - | - |
| ^1^Significance of markers determined using Wilcoxon Mann-Whitney test: *** p<0.01 highly significant, ** p<0.05 significant, * p<0.01 suggestive  LA4152 is a susceptible IBL  HM indicates homozygous LA1589  HET indicates heterozygous LA1589/E-6203  - indicates homozygous E-6203 | | | | | | |

| **Table S5.** Loci and coordinates for previously known tomato genes and LA1589 orthologs involved in resistance to *P. syringae* | | | | | |
| --- | --- | --- | --- | --- | --- |
| **Locus** | **Locus Name** | **Solgenomics locus** | **LA1589 ortholog** | **LA1589 ortholog coordinates** | **LA1589 gene within IBL region?** |
| *FLS2.1* | Flagellin Sensing 2 | Solyc02g070890.3.1 | Spim02g021150.1.1 | Spim0.1ch 02: 41246499-41250461 | No |
| *FLS2.2* | Flagellin Sensing 2 | Solyc02g070910 | Spim02g021180.1.1 | Spim0.1ch02: 41254163-41257963 | No |
| *FLS3* | Flagellin Sensing 3 | Solyc04g009640.2.1 | Spim04g009330.1.1 | Spim0.1ch04: 5202597-5206136 | No |
| *PRF* | Pseudomonas resistance | Solyc05g013280 | Spim05g011870.1.1 | Spim0.1ch05: 5340155-5345742 | No |
| *PTO* | Pto-like serine/threonine kinase | Solyc05g013300.1.1 | Spim05g011900.1.1 | Spim0.1:ch05: 5368138-5369109 | No |

| **Table S6.** Metrics for Illumina whole-genome sequencing | | | | | |
| --- | --- | --- | --- | --- | --- |
| Sample pool^1^ | Mapped to | Genome coverage | Total reads after trimming^2^ | Total reads passing quality filter | Percentage of reads passing BWA-MEM quality filter |
| LA1589 SR | LA1589 | 388X | 1972121919 | 1433302145 | 72.7% |
| LA1589 HS | LA1589 | 349X | 1982856536 | 1286732013 | 64.9% |
| LA1589 SR | Heinz BG-1706 | 397X | 1970656997 | 1466775692 | 74.4% |
| LA1589 HS | Heinz BG-1706 | 358X | 1980465396 | 1319936306 | 66.6% |
| LA1589 SR | LA2093 | 395X | 1970842936 | 1457012516 | 73.9% |
| LA1589 HS | LA2093 | 354X | 1981293878 | 1308252988 | 66.0% |
| ^1^SR is strongly resistant and HS is highly susceptible.  ^2^Reads were trimmed using Cutadapt v2.4 (Martin 2011). | | | | | |
